# Fair positive unlabeled learning for predicting undiagnosed Alzheimer’s disease in diverse electronic health records

**DOI:** 10.1038/s41746-025-02111-1

**Published:** 2025-11-27

**Authors:** Thai Tran, Mingzhou Fu, Jessica Fung, Sriram Sankararaman, David A. Elashoff, Keith Vossel, Timothy S. Chang

**Affiliations:** 1 Department of Neurology, David Geffen School of Medicine, University of California, Los Angeles, CA USA; 2 Medical Informatics Home Area, Department of Bioinformatics, University of California, Los Angeles, CA USA; 3 Computational Medicine, University of California, Los Angeles, CA USA; 4 Department of Biostatistics, University of California, Los Angeles, CA USA

**Keywords:** Dementia, Alzheimer's disease, Machine learning, Predictive markers

## Abstract

Alzheimer’s Disease (AD), the most common neurodegenerative disease, is underdiagnosed and more prominent in underrepresented groups. We performed semi-supervised positive unlabeled learning (SSPUL) coupled with racial bias mitigation for equitable prediction of undiagnosed AD from diverse populations at UCLA Health using electronic health records. SSPUL achieved superior sensitivity (0.77–0.81) and area under the precision recall curve (AUCPR) (0.81–0.87) across non-Hispanic white, non-Hispanic African American, Hispanic Latino, and East Asian groups compared to supervised baseline models (sensitivity: 0.39–0.53; AUCPR: 0.3–0.7). SSPUL also exhibited superior fairness as evidenced by the lowest cumulative parity loss. We identified top shared and distinct features among labeled and unlabeled AD patients, including those that are neurological (e.g., memory loss) and non-neurological (e.g., decubitus ulcer). We validated our results using polygenic risk scores, which were higher in labeled and predicted positives than in predicted negatives among non-Hispanic white, Hispanic Latino, and East Asian groups (*p* < 0.001).

## Introduction

Alzheimer’s Disease (AD) is the most common neurodegenerative disease and the sixth leading cause of death in the United States, affecting 1 in 9 (10.7%) Americans aged 65 and older^1,2^. Currently, more than 6 million Americans are living with AD, and 1 in 3 seniors dies with AD or another dementia^1^. Combined, the total cost of treatment for individuals with AD and other dementias is $345 billion in 2023 and is projected to increase to over $1 trillion by 2050^1,2^.

Coupled with the substantial health and economic burden that AD poses, early AD diagnosis is important for patients to implement lifestyle changes, plan for the future, and receive optimal treatment^3,4^. Previous studies have shown a discrepancy between the prevalence of AD in large longitudinal cohort studies and real-world, community settings^5–7^. While large cohort studies use in-person assessments of dementia (i.e., the gold standard), real-world settings have relied on data from Medicare claims to estimate AD prevalence. Compared to the gold standard diagnoses, the sensitivity of Medicare claims was only 50–65%, highlighting the significant underdiagnosis of AD in real-world settings. In understudied populations, AD underdiagnosis is further exacerbated^8–10^. The estimated AD prevalence based on projections from longitudinal studies was 10% among non-Hispanic whites (NH-whites), 14% among Hispanic Latinos (HLs), 18.6% among non-Hispanic African Americans (NH-AfAms), and 7.4% among East Asians (EA)^11,12^. Despite being almost 2 times more likely to have AD than NH-whites, NH-AfAms are only 34% more likely to have a diagnosis in Medicare claims data^11,13^. Likewise, despite being ~1.5 times more likely to have AD than NH-whites, HLs are only 18% more likely to be diagnosed^11,13^. Moreover, studies have reported that Asian Americans, including EA, face higher risk of under-detection and delayed diagnosis of cognitive impairment compared with NH-whites due to lower awareness of AD risk factors and cultural stigma^10^. Taken together, these findings emphasize the need for not only more sensitive, but also more equitable AD diagnosis.

Recent efforts to improve the diagnose of AD include numerous studies that use data-driven methods to predict the onset of AD^14–19^. A number of these studies have leveraged electronic health records (EHR) data for building machine learning models^14,16,18,19^. For example, Barnes et al. developed and validated eRADAR, a linear supervised learning model that predicts undiagnosed dementia using clinical features (e.g., diagnoses, vital signs, healthcare utilization) from EHR data linked to participants in the Adult Changes in Thought study^14^. Similarly, Akter et al. incorporated additional EHR predictors, including behavioral and other clinical risk factors, and evaluated non-linear supervised learning models (e.g., Gradient-Boosted Trees) to predict AD across multiple time windows^20^. These prior studies showcase the advantage of the large sample size of EHR data to train prediction models. Only some prior studies have addressed the limited sample size of understudied populations and exploited the full range of diagnoses as features (not only those selected by experts)^16,19,21^.

Even fewer studies^22,23^ have focused on algorithmic fairness for preventing disparities in dementia diagnosis accuracy in the EHR with respect to race and ethnicity. These fairness-oriented studies employed a supervised learning framework, which has disadvantages in EHR applications, including the requirement of expensive labels and the possible presence of label bias^24,25^. Further, these studies did not focus on predicting undiagnosed AD and relied primarily on knowledge-driven rather than data-driven feature engineering, limiting the scope of predictive features. In addition, Gianattasio et al. did not include popular machine learning fairness metrics, while Yuan et al. evaluated fairness across algorithms without implementing bias mitigation. These gaps highlight that existing fairness studies in AD in the EHR have yet to simultaneously incorporate unlabeled data, data-driven feature learning, and bias mitigation.

Semi-supervised learning (SSL) is a class of machine learning algorithms that learns from a limited set of labels and unlabeled data. A number of studies have utilized SSL to diagnose AD^26–28^ as it can overcome the expense of manual labeling via domain experts^24^. Protected groups are categories of individuals legally protected against discrimination, including but not limited to sex, race and ethnicity, and religion^29^. Pre-existing labels may be subject to bias (i.e., label bias) as they can be differentially ascertained across these groups^25^. In the case of race and ethnicity, systemic racism, practitioner bias, and lack of healthcare access in underserved communities contribute to the lower prevalence of labels for patients belonging to an underrepresented population^30^. SSL can alleviate label bias by leveraging unlabeled data, which is inexpensive and more abundantly available for understudied populations. Positive unlabeled learning (PUL) is a special case of SSL that learns from labeled positive and unlabeled data^31^. EHR data fits well in the PUL framework as the EHR documents what diseases patients have but not what they do not have. Undiagnosed diseases may be undocumented in the EHR due to providers not entering diagnostic codes or not being aware of patient disease conditions. Therefore, in the EHR, there are labeled patients with documented disease and unlabeled patients who may be cases or controls. Of note, PUL has been successfully applied to a variety of disease diagnosis prediction tasks, including AD diagnosis using imaging data^32–35^. However, to date, PUL has not been implemented in the context of AD diagnosis using real world EHR data, which is more practical for prediction on a larger scale than neuroimaging data as the latter is costly and acquired primarily from symptomatic or high-risk patients^36,37^. Moreover, studies that have utilized PUL for AD diagnosis neither stratify their analyses by protected group nor incorporate bias mitigation approaches for reducing outcome inequities among protected groups. Consequently, the models proposed in these studies may be subject to bias and perform more poorly in protected groups.

In our current study, we aimed to identify patients with undiagnosed AD from NH-white, and understudied populations, including NH-AfAm, HL, and EA. We developed a semi-supervised PUL (SSPUL) framework that leveraged the full range of diagnoses via data-driven feature selection and a large number of patients from understudied populations with unknown AD status (N = 25,327) from UCLA Health EHR. To promote algorithmic fairness, we employed pre-processing racial bias mitigation by assigning positive and negative labels for a subset of unlabeled patients based on race-specific probabilistic criteria, as well as post-processing racial bias mitigation by selecting classification cutoffs that optimize the group benefit equality (GBE) for each racial and ethnic group^38^. Additionally, we highlighted important EHR features, including those that are shared and distinct among labeled and unlabeled AD patients. In our model evaluations, we performed rigorous validation at the phenotype level using diagnoses and medications that serve as proxies for AD and at the genotype level by comparing the polygenic risk scores (PRSs) and *Apolipoprotein E (APOE) ε4* allele count for the predictions of a holdout set. Finally, we showed that SSPUL is robust to proxy label distribution shifts. By optimizing both performance and fairness, we demonstrated that our model could predict AD equitably, outperforming baseline supervised models across multiple discrimination performance and fairness metrics. This is the first study to bridge PUL with racial and ethnic bias mitigation in the context of undiagnosed AD prediction in the EHR. Our findings have implications for improving the identification of undiagnosed AD, reducing racial disparities in AD diagnosis, and guiding clinical decision making.

## Results

### UCLA data discovery repository sample description

**Fig. 1:**
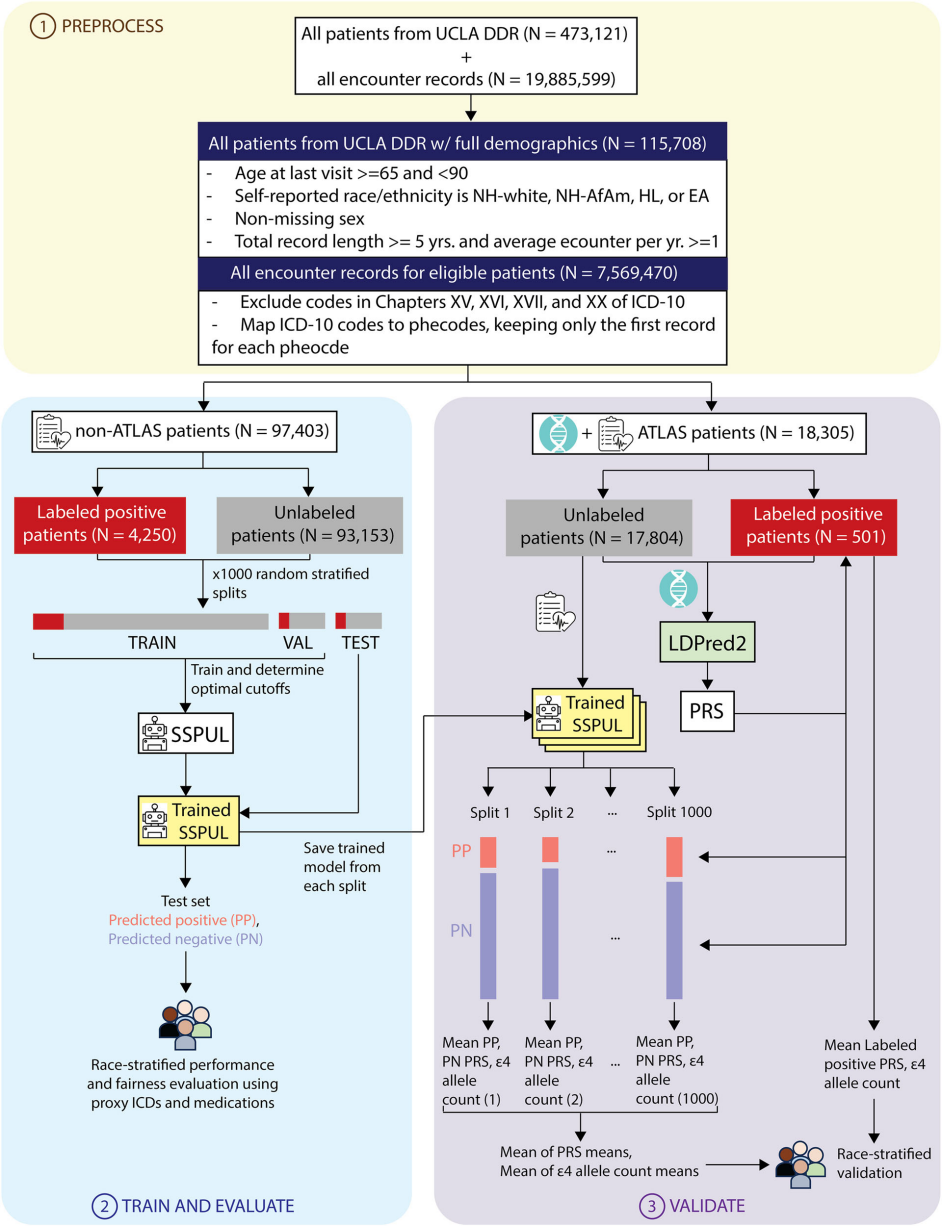
Study design. Phase 1) Patient- and record-level data preprocessing. Phase 2) Train and evaluate. Post-preprocessing, data for non-ATLAS patients was randomly split (labeled positive-stratified). The training set was used for training SSPUL framework, while the validation set was used for determining the optimal GBE cutoff for each race and ethnicity. The trained model was then applied to the test set for race-stratified performance and fairness evaluation using proxy ICDs and medications. Process was repeated 1000 times. Performance and fairness metrics were averaged over the 1000 splits and the trained model from each split was saved for predicting unlabeled ATLAS patients for validation. Phase 3) Validate. Polygenic risk scores were obtained for ATLAS patients using LDPred2. Applying each trained model from phase 2, the mean PRS and ε4 allele count for each final classification of unlabeled patients [i.e., predicted positive (PP) or predicted negative (PN)] was obtained (1000 mean PRS for predicted positives and predicted negatives total). The mean of PRS means and mean of ε4 allele count means were then obtained by aggregating the PRS means and ε4 allele count means, respectively, followed by race-stratified validation. ATLAS = UCLA ATLAS Community Health Initiative, DDR = Data Discovery Repository, EA = East Asian, HL = Hispanic Latino, ICD = International Classification of Diseases, NH-AfAm = Non-Hispanic African American, NH-white = non-Hispanic white, PN=predicted negatives, PP = predicted positives, PRS = polygenic risk scores, SSPUL = semi-supervised positive unlabeled learning, VAL = validation set.

The samples in this study were derived from the UCLA Data Discovery Repository, a de-identified EHR with longitudinal patient records from the UCLA Health System. Samples linked to genetic information collected by the UCLA ATLAS Community Health Initiative (ATLAS)^39^ were used as a holdout set for model validation at the genotype level while non-ATLAS samples were used for training and testing. An overview of the study design is depicted in Fig. 1. Following age and record filtering, we obtained *N* = 129,203 patients. We then excluded those with missing sex, race or ethnicity, resulting in *N* = 115,708 eligible patients (10% missing rate). Non-ATLAS samples consisted of 97,403 patients (Fig. 1 and Table 1). Among labeled positive (LP) and unlabeled patients, we observed statistically different distributions across sex, self-reported NH-AfAm and EA races, record length, number of diagnoses, number of encounters, record density, and age during last visit (*p* < 0.05) (Table 1). Assuming that most unlabeled patients are negative, the higher median record length, number of diagnoses, number of encounters, record density and age during last visit among LP patients are expected. Consistent with previously reported estimates of female AD prevalence^13^, the percentage of female patients diagnosed with AD in our sample was approximately twice that of male patients diagnosed with AD. Compared to the estimated population prevalence of AD over 65 years old among NH-white, NH-AfAm, HL, and EA patients from longitudinal cohort studies^11^ (10%, 18.6%, 14%, and 7.4%, respectively), the AD prevalence in our sample was significantly lower (4.3%, 5.8%, 4.3%, and 3.9%, respectively), indicating that all 4 races and ethnicities were heavily underdiagnosed.

**Table 1:** Distributions of non-ATLAS patient demographics and EHR features, stratified by label (*N* = 97,403)

Characteristic	Overall *N* = 97,403^a^	Labeled positive *N* = 4,250^a^	Unlabeled *N* = 93,153^a^	*p* value^b^
Sex (female)	56,013 (58%)	2757 (65%)	53,256 (57%)	<0.001
Race and ethnicity				
NH-white	70,882 (73%)	3056 (72%)	67,826 (73%)	0.2
NH-AfAm	6003 (6.2%)	350 (8.2%)	5653 (6.1%)	<0.001
HL	10,782 (11%)	468 (11%)	10,314 (11%)	0.9
EA	9,736 (10%)	376 (8.8%)	9,360 (10%)	<0.05
Record length	12 (8, 19)	16 (9, 23)	12 (8, 19)	<0.001
Number of diagnoses	51 (31, 80)	77 (52, 110)	50 (30, 78)	<0.001
Number of encounters	28 (16, 47)	39 (25, 61)	28 (16, 46)	<0.001
Record density (per year)	2.06 (1.42, 3.23)	2.42 (1.59, 3.83)	2.04 (1.41, 3.19)	<0.001
Age at last visit	75 (70, 82)	85 (81, 87)	75 (70, 81)	<0.001

*ATLAS* UCLA ATLAS Community Health Initiative, *EA* East Asian, *HL* Hispanic Latino, *NH-AfAm* non-Hispanic African American, *NH-white* non-Hispanic white.

^a^*n* (%); Median (IQR).

^b^Pearson’s Chi-squared test; Wilcoxon rank sum test.

### SSPUL training set composition and stratified predicted AD prevalence

**Fig. 2:**
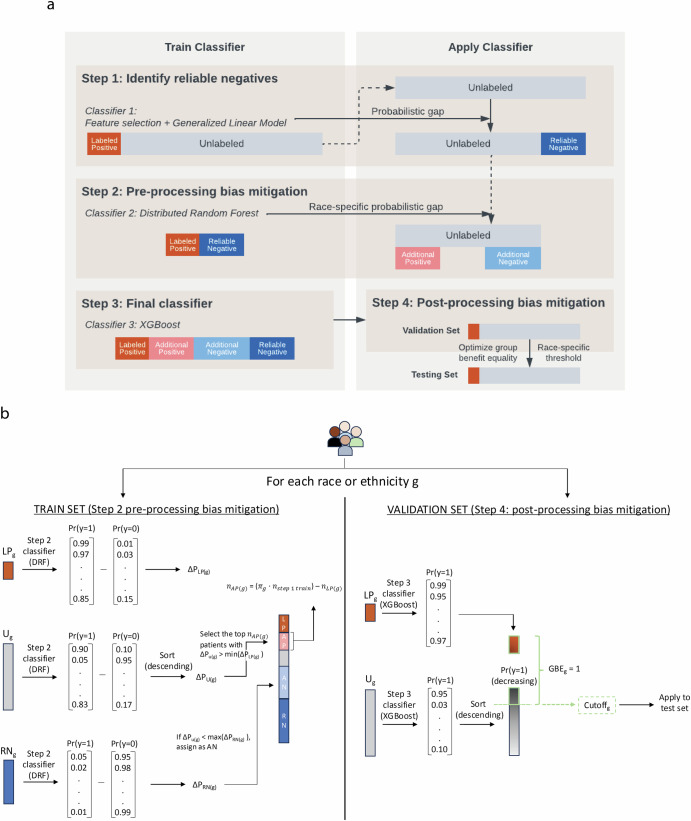
4-step SSPUL framework. **a** SSPUL framework overview. Step 1) Identify reliable negatives: Following feature selection, a Generalized Linear Model (GLM) was trained on labeled positive (LP) and unlabeled data. Reliable negatives were obtained based on having a probabilistic gap that is smaller than the smallest observed probabilistic gap of LPs. Step 2) Pre-processing racial bias mitigation: additional positive (AP) and negative (AN) labels were assigned using race-specific probabilistic gaps. Step 3) Train final classifier: XGBoost classifier was trained on all labeled and pseudo-labeled patients. Step 4) Post-processing bias mitigation: classification cutoffs were determined by optimizing the group benefit equality (GBE) for each race and ethnicity. **b** Pre- and post-processing bias mitigation details. Pre-processing bias mitigation: After training a distributed random forest classifier using LPs and RNs, APs and ANs were assigned for a subset of the remaining unlabeled data such that the following race and ethnic-specific probabilistic criteria are met: 1) APs and ANs have race-specific probabilistic gaps that are greater and smaller than the smallest observed probabilistic gap of LPs and largest observed probabilistic gap of RNs, respectively, for each race and ethnicity; 2) the prevalence of positive labels for each race and ethnicity closely matches the corresponding population AD prevalence. Post-processing bias mitigation: Predicted probabilities for unlabeled patients in the validation set were obtained from the trained final XGBoost classifier. The classification cutoff for each race and ethnicity was determined by optimizing the GBE for each race and ethnicity to ensure that the prevalence of LPs and predicted positives matched that of labeled and proxy-validated positives. The cutoffs were then applied to the test set for classification. AN = additional negative, AP = additional positive, DRF = distributed random forest, g = race or ethnicity variable, GBE = group benefit equality, LP = labeled positive, *n* = number of patients, RN = reliable negative, U = unlabeled, ΔP_LP _= observed probabilistic gap of LPs, ΔP_RN _= observed probabilistic gap of RNs, ΔP_U_=observed probabilistic gap of unlabeled patients, *π*_*g*_ = population prevalence for race or ethnicity g.

We leveraged a 4-step SSPUL framework (Fig. 2a, b) that assigns positive and negative labels to unlabeled training data based on the probabilistic gap and other criteria detailed in Methods and Fig. 2a, b. The probabilistic gap is defined as the difference between the probability of an instance having a label given its features and the complement: ΔPr(x) = Pr(y = 1|x) – Pr(y = 0|x)^31^. Reliable negatives (RNs) were identified in step 1 from unlabeled patients that have a probabilistic gap that is smaller than the smallest observed probabilistic gap of LP patients (ΔP_LP_). To attain racial fairness for our model, we performed pre-processing bias mitigation by assigning additional positive (AP) and negative (AN) labels to a subset of the remaining unlabeled data in the training set. APs were assigned to patients with probabilistic gaps above the smallest ΔP_LP_ for their race or ethnicity. APs were added until APs + LPs matched each group’s population prevalence estimate based on the Chicago Health and Aging Project and meta-analysis^11,12^. ANs were assigned to patients with probabilistic gaps below the largest probabilistic gap of RNs (ΔP_RN_) for their race or ethnicity. In the last step, we implemented post-processing bias mitigation by selecting the classification cutoff for each race and ethnicity to optimize GBE in the validation set, ensuring that prevalence of predicted positives matched that of LPs and proxy-validated positives. The cutoffs were then applied to the test set for classification. For clarity, the cutoff method will be referred to in parentheses [e.g., SSPUL (GBE)].

Together, LPs and pseudo-labels (APs, ANs, and RNs) made up on average 80% of the total training set (95% CI: 69%, 85%), with on average 12.4%, 19.6%, 14.6%, and 9.8% of NH-white, NH-AfAm, HL, and EA positive labels (LP or AP), respectively. In contrast, baseline supervised models had substantially lower mean prevalences of positive labels (4.3%, 5.8%, 4.3%, and 3.9% for NH-white, NH-AfAm, HL, and EA, respectively). Supplementary Fig. 1a shows confusion matrices for APs and ANs, stratified by race and ethnicity. The mean sensitivity of pseudo-labels compared to LPs and proxy-validated positives across 1000 random train/validation/test splits was 83.8%, 92.3%, 89.0%, and 67.4%, while the mean false discovery rate was 4.1%, 6.1%, 7.6%, and 0.8% for NH-white, NH-AfAm, HL, and EA, respectively. Due to the lower population prevalence for EA (7.4%), fewer APs were added compared to other groups, resulting in a lower sensitivity and false discovery rate. RNs and ANs made up on average 87% of the final training set (95% CI: 85%, 88%) for SSPUL, with a mean false omission rate of 1.6%, 1.4%, 1.4%, and 3.6% for NH-white, NH-AfAm, HL, and EA, respectively. Supervised models had a higher prevalence of negative labels (96% of training set) and higher mean false omission rates (15%, 16.7%, 14.6%, and 16.1% for NH-white, NH-AfAm, HL, and EA, respectively). For reference, the mean prevalence of LPs and proxy-validated positives in the training set was 18.7%, 21.5%, 18.3%, and 19.4% for NH-white, NH-AfAm, HL, and EA, respectively. In total, SSPUL (GBE) predicted on average 18.7%, 22%, 18.5%, and 19.3% of NH-white, NH-AfAm, and HL patients to have AD, a significant increase from the labeled AD prevalences, and closely matching the LP and proxy-validated AD prevalences (Supplementary Table 1). In addition, while sex was not optimized for, SSPUL (GBE) predicted higher AD prevalence in females than in males (19.3% and 18.5%, respectively), consistent with established findings^13^ (Supplementary Table 1).

### SSPUL outperforms baseline supervised models in accurately identifying undiagnosed AD patients

Table 2 shows the discrimination performance of SSPUL (GBE) relative to baseline models. As baselines for comparison, we tested 2 models, both of which were trained on noisy negative labels and had cutoffs selected based on maximizing the Matthew’s Correlation Coefficient (MCC) for unlabeled data in the validation set using proxy labels. Supervised (risk factors/MCC) was trained using only demographics and a list of manually curated AD risk factors while Supervised (full/MCC) was trained using all significant features from feature selection. Compared to the baseline models, SSPUL (GBE) performed best across all races and ethnicities with regards to sensitivity (0.77–0.81), balanced accuracy (BA) (0.86–0.88), area under the receiver operating characteristic curve (AUC) (0.91–0.95), and area under the precision recall curve (AUCPR) (0.81–0.87) (*p* adj. < 0.001). Supervised (full/MCC) performed best with regards to precision for NH-white, NH-AfAm, and EA (0.86, 0.83, and 0.88, respectively) (*p* adj. < 0.001). This may be partially attributed to the presence of significantly more negative labels in the training set, and as a result, Supervised (full/MCC) on average making significantly fewer positive predictions (768 [95% CI: 622, 917] vs. 1423 [95% CI: 1345; 1507]) (*p* value adj. < 0.001) and more negative predictions (8548 [95% CI: 8398; 8693] vs. 7,893 [95% CI: 7809; 7970]) (*p* value adj. < 0.001) compared to SSPUL (GBE). For the HL group, Supervised (full/MCC) and SSPUL (GBE) had similar precision (*p* adj. = 1). Supervised (full/MCC) had lower sensitivity across all races and ethnicities (0.39–0.52) (*p* adj. < 0.001 for all comparisons), predicting on average 2 times less positives than SSPUL (GBE) (Supplementary Fig. 1b). Notably, SSPUL (GBE) also outperformed vanilla 2-step PUL^31^ using race-specific GBE cutoffs with regards to sensitivity, precision, and BA for NH-white, NH-AfAm, and HL (*p* adj. < 0.001) (Supplementary Table 2), demonstrating the benefit of training using additional pseudo-labels. A similar trend was observed for EA, although sensitivity did not differ significantly (*p* adj. = 0.07). Overall, SSPUL (GBE) had the best sensitivity/precision tradeoff, marked by a substantial gain in mean sensitivity (+0.25 to +0.38) and relatively marginal loss in precision (0 to −0.11) across all races and ethnicities compared to Supervised (full/MCC).

**Table 2:** Test set performance of SSPUL (GBE) and baseline models

Race/ ethnicity	Model	Sensitivity	Precision	Specificity	B. Accuracy	AUC	AUCPR
NH-white	Supervised (risk factors/MCC)	0.45	0.3	0.8	0.63	0.69	0.3
		(0.27, 0.57)	(0.25, 0.37)	(0.71, 0.92)	(0.59, 0.65)	(0.68, 0.71)	(0.28, 0.33)
	Supervised (full/MCC)	0.45	0.86	0.99	0.72	0.82	0.68
		(0.39, 0.51)	(0.79, 0.92)	(0.98, 0.99)	(0.69, 0.75)	(0.80, 0.84)	(0.65, 0.71)
	SSPUL (GBE)	0.8	0.8	0.96	0.88	0.95	0.87
		(0.71, 0.87)	(0.71, 0.87)	(0.95, 0.98)	(0.83, 0.92)	(0.94, 0.96)	(0.80, 0.91)
NH-AfAm	Supervised (risk factors/MCC)	0.53	0.32	0.76	0.65	0.71	0.34
		(0.33, 0.69)	(0.24, 0.41)	(0.65, 0.89)	(0.59, 0.71)	(0.65, 0.76)	(0.25, 0.43)
	Supervised (full/MCC)	0.51	0.83	0.98	0.75	0.82	0.7
		(0.41, 0.63)	(0.70, 0.94)	(0.95, 0.99)	(0.70, 0.80)	(0.76, 0.87)	(0.62, 0.78)
	SSPUL (GBE)	0.81	0.79	0.96	0.88	0.95	0.88
		(0.64, 0.92)	(0.63, 0.91)	(0.91, 0.98)	(0.79, 0.94)	(0.92, 0.98)	(0.79, 0.94)
HL	Supervised (risk factors/MCC)	0.44	0.29	0.81	0.63	0.69	0.3
		(0.26, 0.59)	(0.23, 0.38)	(0.72, 0.92)	(0.58, 0.68)	(0.65, 0.74)	(0.23, 0.36)
	Supervised (full/MCC)	0.52	0.77	0.97	0.74	0.82	0.68
		(0.42, 0.62)	(0.65, 0.87)	(0.95, 0.99)	(0.70, 0.79)	(0.77, 0.86)	(0.61, 0.74)
	SSPUL (GBE)	0.77	0.77	0.96	0.87	0.95	0.84
		(0.60, 0.89)	(0.61, 0.90)	(0.92, 0.98)	(0.77, 0.93)	(0.92, 0.97)	(0.74, 0.92)
EA	Supervised (risk factors/MCC)	0.42	0.32	0.82	0.62	0.69	0.33
		(0.21, 0.57)	(0.25, 0.43)	(0.73, 0.94)	(0.57, 0.67)	(0.64, 0.73)	(0.26, 0.40)
	Supervised (full/MCC)	0.39	0.88	0.99	0.69	0.79	0.64
		(0.29, 0.49)	(0.78, 0.97)	(0.98, 1)	(0.65, 0.74)	(0.74, 0.84)	(0.57, 0.71)
	SSPUL (GBE)	0.77	0.77	0.96	0.86	0.91	0.81
		(0.67, 0.84)	(0.69, 0.86)	(0.94, 0.98)	(0.81, 0.90)	(0.87, 0.94)	(0.73, 0.87)

Metrics reported are means of 1000 random test sets with 95% CI. Cutoffs for baseline supervised models were selected by maximizing the MCC for unlabeled data in the validation set using proxy labels. Cutoffs for SSPUL was selected by optimizing the GBE for each race/ethnicity in the validation set using positive and proxy labels.

*AUC* area under the curve, *AUCPR* area under the precision recall curve, *B. accuracy* balanced accuracy, *EA* East Asian, *GBE* group benefit equality, *HL* Hispanic Latino, *MCC* Matthew’s correlation coefficient, *NH-AfAm* non-Hispanic African American, *NH-white* non-Hispanic white, *SSPUL* semi-supervised positive unlabeled learning.

**Fig. 3:**
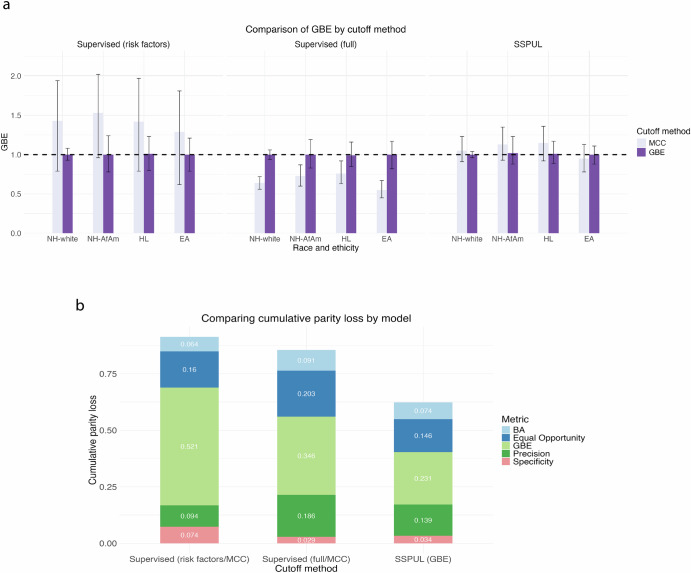
Evaluation of fairness across models. Fairness metrics were averaged over 1000 test sets. **a** Comparison of GBE by cutoff method. **b** Comparison of cumulative parity loss across models. BA balanced accuracy, EA East Asian, GBE group benefit equality, HL Hispanic Latino, MCC Matthew’s Correlation Coefficient, NH-AfAm non-Hispanic African American, NH-white non-Hispanic white, NPV negative predictive value, SSPUL semi-supervised positive unlabeled learning.

We also evaluated discrimination performance metrics if Supervised (full) used GBE rather than MCC, and if SSPUL used MCC instead of GBE to distinguish the benefit of SSPUL and GBE optimization. When we optimized the GBE instead of using MCC to determine cutoffs for all races and ethnicities for Supervised (full), it still under-performed relative to SSPUL (GBE) with regards to sensitivity (0.57–0.62 vs. 0.77–0.81) (*p* adj. < 0.001) despite the adjusted cutoffs yielding more positive predictions overall (Supplementary Table 2). Regardless of the cutoff selection method, SSPUL was superior to supervised learning with regards to discrimination performance. Comparing cutoff methods for SSPUL, we observed race and ethnicity-specific sensitivity/precision tradeoffs (Supplementary Table 2). For NH-white, SSPUL (MCC) provided a modest gain in sensitivity (+5%) without loss in precision, while EA showed only minor changes (+1% sensitivity, –5% precision). Consistent with these patterns, the mean GBE for NH-white (1.05) and EA (0.95) using the MCC cutoff were close to those under the GBE cutoff (both 1), whereas greater discrepancies were observed for NH-AfAm and HL (Fig. 3a). Despite these tradeoffs, GBE optimization consistently yielded the best balance between sensitivity and precision across all models (Table 2, Supplementary Table 2).

In addition to having improved discrimination performance, we showed that SSPUL achieved the best calibration performance relative to baseline models (Supplementary Fig. 2a). Calibration performance indicates the quality of model predictions with respect to proxy-validated labels, allowing predicted probabilities to be interpreted as long-run frequencies^40,41^. For example, if a model’s predicted probability for a group (bin) of patients to have AD were 10%, then we would expect 10% of patients in that group to have AD. Each bin in Supplementary Fig. 2a represents approximately 10% mean predicted probability. While model over-confidence was evident for parts of the SSPUL calibration curve (i.e., points in bins 7–9), overall, SSPUL achieved the lowest expected calibration error among models, indicating the best calibration performance (Supplementary Fig. 2a). In contrast, the calibration curve for Supervised (full) suggests that the model was highly under-confident for almost all patients above the first bin. We observed a similar, but less pronounced, pattern for Supervised (risk factors). Consistent with these findings, Supplementary Fig. 2b shows that most predicted probabilities of SSPUL for proxy-validated AD cases for an example test set were 0.9–1 while most predicted probabilities of the baseline models fell in the lower quartile. Moreover, this was reflected in SSPUL’s significantly lower balanced Brier score (0.17–0.25 vs. 0.8–0.88) and positive Brier score (0.09–0.2 vs. 0.8–0.88) compared to those of baseline models across all races and ethnicities (*p* adj. < 0.001) (Supplementary Fig. 2c).

### SSPUL achieves racial fairness without compromising discrimination performance

In addition to discrimination performance, we compared fairness across models by measuring the differences between an unprivileged group (NH-AfAm, HL, or EA) and the privileged group (NH-white) in predicting undiagnosed AD with respect to discrimination performance metrics (Eq. 4). We also aggregated the absolute differences across unprivileged groups for each metric to obtain metric-specific parity losses (Eq. 7), which were then summed to obtain the cumulative parity loss (Eq. 8)^42^. SSPUL (GBE) was the fairest relative to baseline models as evidenced by the lowest cumulative parity loss (*p* adj. < 0.001) (Fig. 3b). Stratifying by metric, SSPUL (GBE) had lower mean BA and mean precision parity losses (0.074 and 0.139, respectively) than Supervised (full/MCC) (0.091 and 0.186, respectively) (*p* adj. < 0.001). Compared to Supervised (risk factors/MCC), SSPUL (GBE) had lower mean specificity parity loss (0.034 vs. 0.074) but higher mean BA parity loss (0.074 vs. 0.064) (*p* adj. < 0.001). Compared to both baseline models, SSPUL (GBE) achieved the lowest mean equal opportunity (EO) and mean GBE parity losses (Fig. 3b), suggesting consistent improvement in fairness by narrowing group-level differences in sensitivity and predicted AD prevalence between unprivileged and privileged groups. Honing into group differences underlying parity loss for each metric, SSPUL (GBE) showed the lowest or second lowest absolute mean differences across multiple metrics for all unprivileged/privileged comparisons (Table 3). The most striking absolute mean differences were in EO and GBE between NH-AfAm and NH-white. While SSPUL (GBE) had absolute mean EO and GBE differences of 0 and 0.03, respectively, Supervised (risk factors/MCC) had absolute mean differences of 0.08 and 0.16, and Supervised (full/MCC) had absolute mean differences of 0.06 and 0.1 (*p* adj. < 0.001).

**Table 3:** Test set fairness of SSPUL (GBE) and baseline models

Metric	NH-AfAm vs. NH-white			HL vs. NH-white		
	Supervised (risk factors/MCC)	Supervised (full/MCC)	SSPUL (GBE)	Supervised (risk factors/MCC)	Supervised (full/MCC)	SSPUL (GBE)
BA	0.02	0.03	0	0	0.02	−0.02
	(−0.03, 0.08)	(−0.03, 0.08)	(−0.06, 0.04)	(−0.04, 0.04)	(−0.02, 0.07)	(−0.07, 0.03)
EO	0.08	0.06	0	−0.01	0.06	−0.03
	(−0.02, 0.19)	(−0.04, 0.18)	(−0.12, 0.12)	(−0.09, 0.08)	(−0.03, 0.16)	(−0.13, 0.07)
Precision	0.02	−0.03	−0.01	0	−0.1	−0.04
	(−0.06, 0.10)	(−0.14, 0.07)	(−0.13, 0.08)	(−0.07, 0.06)	(−0.19, 0.00)	(−0.13, 0.06)
Specificity	−0.04	−0.01	−0.01	0.01	−0.01	−0.01
	(−0.08, 0.00)	(−0.03, 0.01)	(−0.05, 0.01)	(−0.02, 0.04)	(−0.03, 0.00)	(−0.03, 0.01)
GBE	0.16	0.1	0.03	−0.01	0.15	0.01
	(−0.21, 0.56)	(−0.05, 0.26)	(−0.17, 0.31)	(−0.28, 0.30)	(0.02, 0.31)	(−0.15, 0.25)

Metric	EA vs. NH-white		
	Supervised (risk factors/MCC)	Supervised (full/MCC)	SSPUL (GBE)
BA	−0.01	−0.03	−0.02
	(−0.05, 0.04)	(−0.07, 0.01)	(−0.07, 0.04)
EO	−0.03	−0.07	−0.03
	(−0.11, 0.06)	(−0.15, 0.03)	(−0.13, 0.08)
Precision	0.02	0.02	−0.03
	(−0.04, 0.10)	(−0.06, 0.10)	(−0.12, 0.07)
Specificity	0.02	0	−0.01
	(−0.02, 0.05)	(−0.01, 0.01)	(−0.03, 0.01)
GBE	−0.2	−0.09	0
	(−0.46, 0.06)	(−0.20, 0.03)	(−0.17, 0.14)

Metrics reported are means of differences between an unprivileged group (NH-AfAm, HL, or EA) and the privileged group (NH-white) for 1000 random test sets with 95% CI. Cutoffs for baseline supervised models were selected by maximizing the MCC for unlabeled data in the validation set using proxy labels. Cutoff for SSPUL model was selected by optimizing the GBE for each race/ethnicity in the validation set using positive and proxy labels.

*BA* balanced accuracy, *EA* East Asian, *EO* equal opportunity, *GBE* group benefit equality, *HL* Hispanic Latino, *NH-AfAm* non-Hispanic African American, *NH-white* non-Hispanic white.

To evaluate whether our pseudo-labeling strategy promoted fairness as a pre-processing bias mitigation approach, we conducted a sensitivity analysis independent of post-processing cutoff selection (Supplementary Table 3). We recoded each race and ethnicity as another race and ethnicity (e.g., NH-white to NH-AfAm) while keeping all other features fixed in the test set, then applied the original classification cutoff to obtain updated sensitives for each model. For example, for models that used GBE cutoffs, the optimal GBE cutoff for NH-whites prior to recoding would be applied to NH-whites that were recoded to NH-AfAms. We observed minimal changes to the sensitivity of the original race and ethnicity for SSPUL (GBE) after recoding (maximum change of 3%). In contrast, we observed larger changes for Supervised (risk factors/MCC) (e.g., recoding NH-AfAm as EA decreased the sensitivity of the former on average by 10%) and Supervised (full/MCC) (e.g., recoding HL as EA decreased the sensitivity of the former on average by 9%). These findings suggest that SSPUL, independent of the cutoff method, exhibited less racial bias than baseline models, as predicted AD probabilities given identical features aside from race and ethnicity remained stable after recoding, leading to minimal sensitivity shifts.

Through post-processing bias mitigation, we further improved fairness by selecting classification cutoffs that optimized the GBE for each race and ethnicity in the validation set. This approach consistently achieved a mean GBE of 1 for all races and ethnicities across all models (Fig. 3a). The Supervised (risk factors/MCC), on the other hand, over-predicted with a mean GBE of 1.43, 1.53, 1.42, and 1.29 while Supervised (full/MCC) under-predicted with a mean GBE of 0.64, 0.73, 0.76, and 0.55 for NH-white, NH-AfAm, HL, and EA, respectively. SSPUL (MCC) slightly over-predicted for NH-white (1.05), NH-AfAm (1.13), and HL (1.15), and under-predicted for EA (0.95). Selecting the cutoff by optimizing the GBE also improved the cumulative parity loss across all models (Supplementary Fig. 3). The parity losses for EO and GBE for all models using GBE cutoffs were lower than those using the MCC cutoff (*p* adj. < 0.001) (Supplementary Fig. 3). Additionally, BA parity loss decreased for the baseline models and specificity parity loss decreased for SSPUL (*p* adj. < 0.001). Racial group differences for each metric for models using the maximum MCC cutoff versus those using the optimal GBE cutoff are reported in Supplementary Tables 4–6.

### Top Predictive Features

**Fig. 4:**
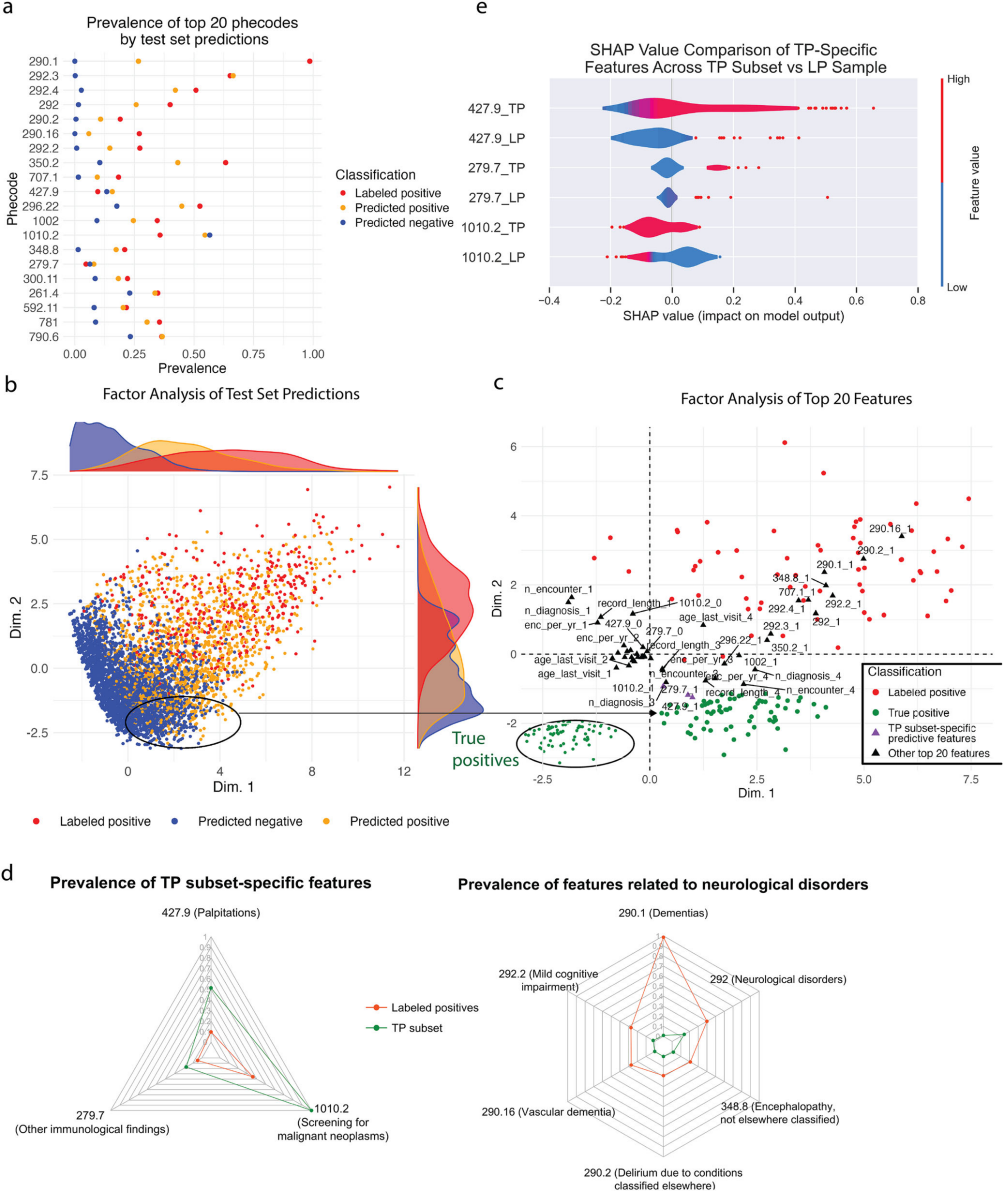
Analyses of top predictive features and test set predictions. **a** Prevalence of top 20 predictive phecodes by test set predictions. **b** Visualization of labeled positives and predicted labels in a 2-dimensional Euclidean feature space via factor analysis. **c** Factor analysis of top 20 predictive features reveals predictive features that are distinct among unlabeled AD patients (purple). Unlabeled AD patients are represented by a true positive (TP) subset (i.e., proxy-validated predicted positives) with coordinates that did not overlap with any of those of LPs in the feature space from **b**. “_1” indicates the positive class of the feature while “_0” indicates the negative class. Age during last visit, number of encounters, number of diagnoses, record density, and record length were discretized into quartiles. **d** Prevalence of top TP subset-specific and neurological features among LPs and the TP subset. **e** Comparison of TP subset-specific features’ impact on TP subset vs. LP sample (N = 215 for each group). TP subset-specific features are binary (0 = low/blue, 1 = high/red). Purple represents a mixture of 0 and 1 values. Dim dimension, EF ejection fraction, LP labeled positive, SHAP SHapley Additive exPlanations, TP True positive.

To infer meaningful phecodes and EHR features for AD prediction, we extracted the top 20 features from the final XGBoost classifier, averaged over 1000 splits (Table 4). Among these features, 13 were related to mental or neurological disorders (e.g., memory loss) and 5 were related to age, number of diagnoses, or healthcare utilization (e.g., number of encounters). Other features included screening for malignant neoplasms (1010.2) and decubitus ulcer (707.1). 4 of the top 20 overlapped with phecodes that map to proxy ICDs. Consistent with their importance for AD prediction, most of the top features had a higher prevalence in LPs and predicted positives compared to predicted negatives in an example test set (Fig. 4a). In fact, the top features related to neurological or mental disorders (290.1, 292.3, 292.4, 292, 290.2, 290.16, 292.2, and 348.8) were almost absent in predicted negatives. Taking the top 20 features, we performed Factor Analysis of Mixed Data (FAMD)^43^ on the predictions of the same test set in Fig. 4a to visualize them in a 2-dimensional Euclidean space (Fig. 4b). Plotting their coordinates along the first and second dimensions, we observed LPs and predicted positives to form overlapping clusters that are mostly distinct from that of predicted negatives, indicating clear separability with respect to the prediction labels based on the top predictive features of the model.

**Table 4:** Variables of importance of final XGBoost classifier

Feature	Scaled importance	Description	Group
290.1	0.964987329	Dementias	Mental disorders
292.3	0.638804372	Memory loss	Mental disorders
292.4	0.290348042	Altered mental status	Mental disorders
292	0.145857822	Neurological disorders	Mental disorders
292.2	0.041186049	Mild cognitive impairment	Mental disorders
350.2	0.022679351	Abnormality of gait	Neurological
Age last visit	0.021279103	Age last visit	Demographics
290.2	0.013784367	Delirium due to conditions classified elsewhere	Mental disorders
290.16	0.012180045	Vascular dementia	Mental disorders
291.4	0.006907559	Specific nonpsychotic mental disorders due to brain damage	Mental disorders
292.1	0.004888427	Aphasia/speech disturbance	Mental disorders
Number of encounters	0.004420907	Number of encounters	Healthcare utilization
Record density (per year)	0.003414709	Record density (per year)	Healthcare utilization
292.5	0.003343616	Transient alteration of awareness	Mental disorders
348.8	0.003221973	Encephalopathy, not elsewhere classified	Neurological
Record length	0.002834423	Record length	Healthcare utilization
Number of diagnoses	0.002739678	Number of diagnoses	Comorbidity burden
1010.2	0.002588041	Screening for malignant neoplasms	Cancer screening
296.22	0.002113258	Major depressive disorder	Mental disorders
707.1	0.001790243	Decubitus ulcer	Dermatologic

Features are sorted in order of decreasing scaled importance, which reflects each feature’s overall contribution to improving model accuracy when it is used to split a tree node. Scaled importance is averaged over 1000 random splits.

Given the substantial contribution of the top 20 features in driving model predictions, it is important to assess whether their influence is consistent across racial and ethnic groups. Disparities in feature importance may indicate model bias and compromise group fairness^44^. To assess whether SSPUL relies on the top predictive features differently across racial and ethnic groups, we examined whether the magnitude and/or direction of the top 20 features’ SHAP values^45^ differed between the privileged group (NH-white) and unprivileged groups (NH-AfAm, HL, EA) in a random test set.

We quantified the magnitude of feature influence by averaging the absolute SHAP values of each top feature within each racial and ethnic group. For most top features, absolute SHAP values did not differ significantly between NH-white and unprivileged groups (Mann–Whitney U test with Bonferroni correction, *p* adj. > 0.05). Exceptions included screening for malignant neoplasms (1010.2), record length, and other immunological findings (279.7). In addition to the magnitude, we analyzed the correlation between each feature’s value and its corresponding SHAP value within each racial and ethnic group and tested whether these correlations differed significantly across NH-white and unprivileged groups. Across all comparisons, we found no statistically significant differences in correlation [NH-white vs. NH-AfAm (*p* = 0.84), NH-white vs. HL (*p* = 0.43), NH-white vs. EA (*p* = 0.2)]. Taken together, these findings suggest no meaningful variation in either SHAP directionality or magnitude across racial and ethnic groups, reinforcing the fairness of our model’s predictions. Supplementary Fig. 4 visually illustrates the similarity in the direction and magnitude of SHAP values across NH-white, NH-Afm, HL, and EA groups.

### Predictive features corresponding to undiagnosed AD

Given the limited number of positive labels that are selected at random, a unique objective of PUL is to predict positives that are feature-wise distinct from LPs in the training or test set in order to obtain a positive sample that more closely resembles the true positive (TP) distribution. To do this, we performed FAMD using the top 20 predictive features and selected a subset of TPs (i.e., proxy-validated predicted positives) from the output feature space (Fig. 4b) with coordinates that do not overlap with those of LPs (except one outlier). We then discretized age during last visit, number of encounters, record density, record length, and number of diagnoses. Subsequently, we re-ran FAMD to obtain coordinates of the projections on the categories of each feature on the feature space from the first FAMD run (Fig. 4c). As expected, both LPs and the TP subset formed clusters that deviated from points representing the negative class of the top 20 predictive features (i.e., the black triangles clustered around the origin in Fig. 4c). While the coordinates of LPs overlapped with those corresponding to the positive class of top neurological predictive features [e.g., 290.1 (dementias), 292 (neurological disorders), 348.8 (encephalopathy, not elsewhere classified), 290.2 (delirium due to conditions classified elsewhere), 290.16 (vascular dementia), 292.2 (mild cognitive impairment)], the coordinates of the TP subset were closer to those corresponding to the positive class of top non-neurological predictive features [427.9 (palpitations), 1010.2 (screening for malignant neoplasms), 279.7 (other immunological findings)].

To further evaluate these findings, we compared the prevalence of the TP subset-specific features with those overlapping with LPs in the test set (Fig. 4d). We observed a higher prevalence of top non-neurological features in the TP subset relative to LPs (*p* adj. < 0.01 for all features). On the contrary, we observed a lower prevalence of top neurological features in the TP subset relative to LPs (*p* adj. < 0.001 for all features). Additionally, we found that 427.9 and 279.7 had a greater impact on the model output of the TP subset compared to that of LPs as evidenced by a higher mean absolute SHAP value across all features (*p* adj. < 0.001) (Fig. 4e). Although there was no significant difference in mean absolute SHAP values for screening for malignant neoplasms (1010.2), the proportion of patients with positive SHAP values for 1010.2 differed significantly between the TP and LP subsets (Fisher’s exact test, *p* < 0.001). While 1010.2 contributed both positively and negatively to model predictions in the TP subset, it had a predominantly negative effect in the LP subset.

### Genotype validation of holdout set predictions

**Fig. 5:**
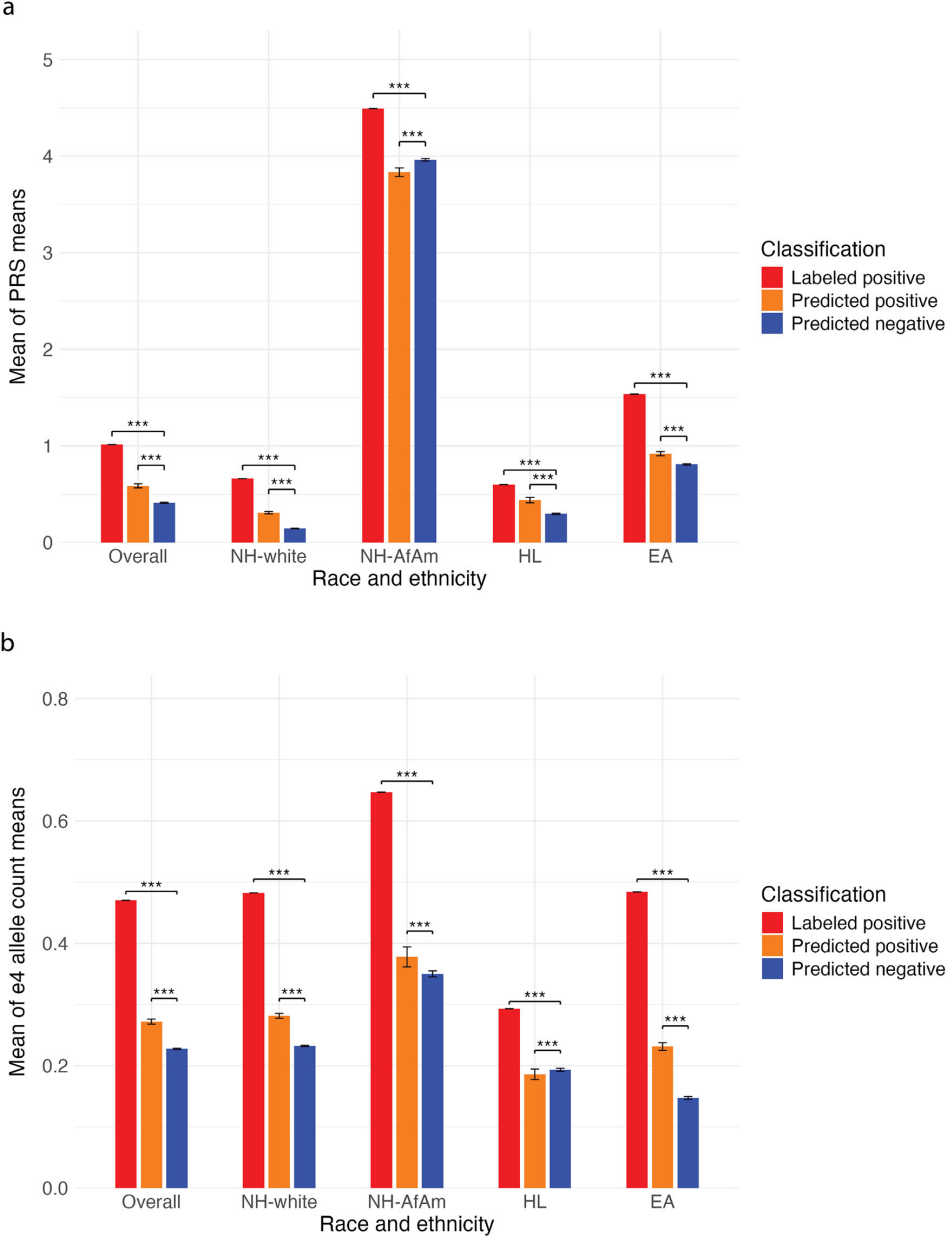
Genotype validation of holdout set prediction labels. Prediction labels for the holdout set were inferred using 1000 trained models. The mean polygenic risk scores (PRSs) and ε4 allele counts were obtained for LPs and each prediction label for each iteration, then averaged. **a** PRSs, stratified by holdout set prediction labels. **b** ε4 allele count, stratified by holdout set prediction labels. EA East Asian, HL Hispanic Latino, NH-AfAm non-Hispanic African American, NH-white non-Hispanic white, PRS polygenic risk score.

Genetic predisposition plays an important role in AD^46,47^, with more than 40 genetic risk loci identified^48^. For this reason, we also validated our model’s predictions on a holdout test set containing ATLAS patients using polygenic risk scores (PRS) calculated using a late-onset AD GWAS^49^ (Fig. 5a). To do this, we applied each final SSPUL classifier trained on one of 1000 random splits to the holdout set. We measured the mean PRS of predicted positives or predicted negatives from each split, and then aggregated them to obtain the mean of PRS means. LPs had a single PRS mean as they were independent of the model’s predictions. Overall, we observed the mean PRS of LPs (1.01) and the mean of PRS means of predicted positives (0.59 [95% CI: 0.55, 0.64]) to be significantly higher than the mean of PRS means of predicted negatives (0.41 [95% CI: 0.40, 0.42]) (p adj. < 0.001). Stratifying our analysis by race and ethnicity, we observed a similar trend for NH-white and HL, but not for NH-AfAm. In the NH-AfAm group, the mean of PRS means of predicted positives (3.83 [95% CI: 3.76, 3.94]) was slightly lower than that of predicted negatives (3.96 [95% CI: 3.93, 3.98]) (p adj. < 0.001). This observation may partially be explained by the lack of generalizability of PRSs calculated from European subjects^50,51^.

We also performed a similar analysis comparing the mean of the *Apolipoprotein E (APOE) ε4* allele, a significant contributor to AD risk^47,48^, count means (Fig. 5b). For NH-white, NH-AfAm, and EA, we observed the mean ε4 allele count of LPs and the mean of ε4 allele count means of predicted positives to be significantly higher than the mean of ε4 allele count means of predicted negatives (*p* adj. < 0.001). Consistent with these findings, the mean proportion of patients with at least one ε4 allele count was higher among LPs and predicted positives than among predicted negatives overall, and within NH-white and EA (*p* < 0.001) (Supplementary Fig. 5a). The proportion of NH-AfAm or HL patients with at least one ε4 allele count was not significantly different among predicted positives and predicted negatives (*p* = 1, 0.99 for NH-AfAm and HL comparisons, respectively). In addition to the slightly lower mean ε4 allele count observed in predicted positives relative to predicted negatives for HL, this discrepancy may partly be attributed to the lower AD risk of *APOE-ε4* in NH-AfAms and HLs compared to NH-whites^52^.

We also measured the mean PRS and mean ε4 allele count, stratified by race and ethnicity, for proxy-validated labels and compared them with the mean of PRS means and mean of ε4 allele count means for our model’s predictions (Supplementary Fig. 5b, c). We observed similar trends for both mean PRS and mean ε4 allele count for proxy-validated positives and proxy-validated negatives across all races and ethnicities, further supporting our model’s predictions at the genotype level. The differences between proxy-validated positives and proxy-validated negatives were not significant for mean ε4 allele count among NH-AfAm and HL, nor for mean PRS among NH-AfAm, HL, and EA. These results are likely due to these groups having smaller sample sizes compared to NH-white. In addition, the mean of ε4 allele count findings may be driven by the lower *APOE-ε4* AD risk in NH-AfAm and HL, as previously mentioned^52^.

### SSPUL demonstrates robustness to proxy label distribution shifts

We leveraged proxy labels to select the best-performing final classifier of SSPUL (GBE) and optimized classification cutoffs using race-specific GBE. While necessary for model selection and tuning in the absence of gold standard labels, this could unintentionally bias SSPUL (GBE) toward learning the defined proxy label distribution. To test the robustness of our model to shifts in the proxy label distribution, we conducted a sensitivity analysis using different proxy subsets (detailed in “Methods”). Across nearly all subsets, mean sensitivity and precision remained stable across all racial and ethnic groups (Supplementary Table 7). Excluding subsets involving F03.90 (F03.90, 290.1, random5) and R41.3 (R41.3, 292.3, random1), the mean sensitivity and precision ranged from 0.75–0.81 and 0.76–0.82, respectively. These ranges were comparable to those obtained using the full proxy definition (0.77–0.81 for sensitivity and 0.77–0.8 for precision).

Removal of subsets involving F03.90 or R41.3 was more extreme as these ICD codes were the most common sole proxies, accounting for 6.1% and 44.6% of all validated cases (excluding labeled positives), respectively. This led to a significant decrease in the estimated AD diagnosis and proxy prevalence across races and ethnicities and corresponding shifts in GBE-optimized cutoffs. As a result, precision decreased (0.72–0.76 and 0.53–0.67 for subsets involving F03.90 and R41.3, respectively) because some predictions that would have been true positives under the full proxy definition were reclassified as false positives. Sensitivity also decreased (0.71–0.75 and 0.59–0.68 for subsets involving F03.90 and R41.3, respectively) due to a stricter cutoff classifying more negatives, some of which were false.

These findings suggest that SSPUL (GBE) is generally robust to shifts in proxy label definitions. Mean sensitivity and precision remained consistent across a wide range of proxy subsets, with notable drops only in cases where the excluded proxies were the only ones validating a substantial number of positive cases. However, the exclusion of such proxies is an extreme circumstance.

## Discussion

In this study, we developed SSPUL for equitably and accurately identifying undiagnosed AD given a limited number of positive labels. The innovation of our study lies in coupling a PUL framework with pre- and post-processing bias mitigation approaches via race-specific probabilistic criteria and optimal GBE thresholding. We addressed the limitations of label bias and expense of supervised learning employed in previous studies on fairness in AD^22–25^. Further, we addressed the limitations of previous SSL studies that relied solely on neuroimaging data, which is less practical for large-scale prediction tasks^36,37^. We improved racial group fairness and provided rigorous validation for our results using proxy AD ICD codes, AD medications, and PRSs. To promote interpretability of our model and results, we provided thorough explanations via feature, sensitivity, and SHAP analyses. Finally, we demonstrated the robustness of our model to proxy label distribution shifts.

Our results showed that SSPUL (GBE or MCC) outperformed baseline models with respect to multiple discrimination and calibration performance metrics, most notably sensitivity and AUCPR across NH-white, NH-AfAm, HL, and EA groups. Despite its high discrimination and calibration performances, SSPUL did not suffer with regards to fairness, achieving the lowest cumulative parity loss compared to baseline models. In addition, we showed that the mean sensitivity and precision were stable across a wide range of proxy label definitions, highlighting SSPUL’s robustness. In our feature analysis, we identified top predictive features related to neurological/mental disorders (e.g., memory loss) and non-neurological disorders (e.g., decubitus ulcer). We also identified top predictive features that are specific to undiagnosed AD patients (e.g., palpitations). All top predictive features contributed similarly to AD prediction, both in magnitude and direction, across racial and ethnic groups. Stratifying by prediction labels and race and ethnicity, we found that PRSs were significantly higher for LPs and predicted positives than for predicted negatives in NH-white, HL, and EA patients, while ε4 allele counts were higher in NH-white and EA patients, providing additional support for our model’s predictions.

Among the numerous approaches for leveraging PUL, we applied a 4-step method, which was modified from the vanilla 2-step PUL framework^31^. Other PUL methods such as biased learning rely on the stringent selected completely at random assumption, which states that labeled instances are an i.i.d. sample of the positive distribution^31^. Our data likely does not satisfy this assumption as factors such as systemic biases and access to care shape who receives an AD diagnosis^6,53^. As a result, diagnosed patients are a biased sample of the true positive distribution. We showed that our framework was superior to baseline supervised models and the vanilla 2-step PUL framework. Although supervised models that treat unlabeled instances as negative are commonly used in practice for PUL^31,54^, we demonstrated that they were inferior to SSPUL (GBE) with regards to almost all discrimination and calibration performance metrics across all races and ethnicities. The sensitivity/precision tradeoff for SSPUL (GBE) was significantly more favorable than that for Supervised (full/MCC), especially in HL, where SSPUL (GBE) had on average 25% higher sensitivity without loss in precision. Additionally, we demonstrated that SSPUL (GBE) outperformed vanilla 2-step PUL using race-specific GBE cutoffs, achieving higher precision and balanced accuracy across all racial and ethnic groups, and higher sensitivity among NH-white, NH-AfAm, and HL. These results highlight the effectiveness of the pseudo-labeling strategy in the third step of our framework.

Group fairness adopts various definitions^55^. Here, we defined group fairness based on parity of performance metrics across races and ethnicities as it enabled clear interpretation of our results and is widely used in the literature^22,23,56^. Due to differences in the prevalence of AD among NH-White, NH-AfAm, HL, and EA groups in the EHR and population^11^, we did not deem demographic parity, though popular, appropriate for comparing fairness in our study as it unrealistically favored an equal positive selection rate among all races and ethnicities. Instead, we included GBE as a fairness metric and optimization target for cutoff selection to ensure that the prevalence of each race and ethnicity was reached. GBE is related to the concept of the class prior (the proportion of positive data) in PUL^31^ as the latter must be estimated in order to optimize the former. In our study, we used AD diagnosis and medication proxies to estimate the class prior. By optimizing the GBE for each race and ethnicity, SSPUL (GBE) not only had the lowest cumulative parity loss relative to baseline models, but also the best balance between sensitivity and precision. We observed similar patterns when comparing the MCC cutoff to the GBE cutoffs across models. While GBE optimization yielded an ideal GBE of 1 for most splits, GBE variability existed across models. This variability may be driven by differences in the GBE between the validation and test sets across splits. The relatively high GBE variability for Supervised (risk factors/MCC) may stem from the model’s limited feature set. This limitation may result in many positives and negatives to have similar predicted probabilities, leading to an unstable MCC cutoff across splits.

In addition to GBE optimization, we attribute the superior fairness of our model to our approach of using race-specific population prevalence and ΔP_LP_ and ΔP_RN_ to inform the assignment of pseudo-labels in the semi-supervised framework. This approach produced pseudo-labels that have low false discovery and false omission rates across all racial and ethnic groups and increased the representation of unprivileged races and ethnicities in the training set. The positive impact of these contributions on our model was reflected in our sensitivity analysis of self-reported race and ethnicity features, which showed minimal changes to the sensitivity of each race and ethnicity after recoding it as another race and ethnicity while keeping all other features fixed.

Overall, the top 20 predictive features contributed similarly to AD prediction across all racial and ethnic groups, suggesting that SSPUL learned consistent and generalizable patterns. As expected, 18 of the features were related to mental or neurological disorders, healthcare utilization, or age. Among the remaining features, screening for malignant neoplasms showed a negative association with AD, consistent with prior studies reporting an inverse relationship between AD and cancer^57–59^. Decubitus ulcers have been linked to AD, likely through increased immobility from cognitive and functional decline^60^. More locally, we identified predictive features with low prevalence among diagnosed AD patients, but higher prevalence in a TP subset that is distinct from LPs. These included palpitations (427.9), other immunological findings (279.7), and again, screening for malignant neoplasms. Palpitations may be a risk factor for atrial fibrillation, which has been associated with AD cognitive decline^61^. Other immunological findings include the ICDs abnormal immunological finding in serum (R76.9), other specified abnormal immunological findings in serum (R76.8), and raised antibody titer (R76.0). These may reflect the growing evidence of immune dysregulation in AD^62^.

For training and validation, we leveraged large, diverse EHR data containing rich diagnoses and genetic information derived from the UCLA DDR. To ensure data integrity, we excluded patients with missing demographics. While imputation of missing demographics such as race and ethnicity can be performed to reduce the number of excluded patients, we did not want our assessment of fairness across racial and ethnic groups to be affected or biased by imputation quality. Validated methods for imputation from de-identified EHR have been developed such as the Medicare Bayesian Improved Surname Geocoding, which uses surnames to perform imputation^63,64^. However, such methods are specific to Medicare data elements and we do not have access to names in our de-identified data.

This study has limitations. First, although proxy clinical measures were carefully selected by a domain expert and have been used as silver standards to evaluate unlabeled cases predicted by SSL in a previous study^65^, they are not gold standard ground truths. In the present study, we leveraged proxy ICDs and medications to evaluate model performance and estimate the class prior, which could both be affected by false labels. In future work, we aim to validate our model using gold standard chart review. Second, we were unable to consider group fairness with respect to other protected attributes that are relevant to AD such as rurality and sex. Given the lower prevalence of AD and related dementias in rural counties relative to urban counties^66^, and in males relative to females^13^, future work should incorporate GBE with respect to rurality and sex as additional fairness constraints for optimization^67^. Third, we used a European GWAS for generating PRSs for self-reported NH-AfAm, HL, and EA patients. Though well-powered, it may have resulted in non-optimal risk scores stemming from unaccounted variations in linkage disequilibrium and differences allele frequencies^51^. Previously, we had used trans-ancestry and African American GWAS for genetic risk modeling^50^. However, the purpose of this study was not to employ PRSs to optimize genetic risk prediction itself, but rather to validate our model as a secondary benchmark. To ensure consistency in comparison across racial and ethnic groups, we therefore derived all PRSs from a single GWAS. Nevertheless, we acknowledge that leveraging large GWAS from genetic ancestry specific groups is needed to evaluate genetic ancestry specific PRSs more accurately. This approach could provide stronger validation for our model’s predictions in future studies, particularly for NH-AfAm patients. In addition to addressing these limitations, an important step toward realizing clinical utility is to test the generalizability of our model. We will do this by prospectively validating it in partnering health systems. Given demographic differences across health systems, validating our model outside of UCLA Health would provide stronger evidence of its predictive performance and fairness in mitigating bias.

Overall, we successfully predicted undiagnosed AD from the EHR with high sensitivity and precision by bridging a PUL framework with pre- and post-processing bias mitigation approaches. The implications of this study are 3-fold. First, our model shows potential for assisting providers in identifying high risk AD patients that may be appropriate for further clinical evaluation or screening. Second, by ensuring equitable predictions across racial and ethnic groups, our model can help remedy significant underdiagnosis in underrepresented populations, addressing long-standing disparities in AD diagnosis stemming from systemic and algorithmic biases^9,22^. Lastly, by enhancing the interpretability of our model using a variety of race and ethnicity-stratified analyses, we avoided black box models that could be used to guide clinical decision making.

## Methods

### Data sources

Patient data in this study were derived from the UCLA Data Discovery Repository (DDR), a de-identified EHR containing longitudinal records of patients enrolled in the UCLA Health System, including demographics, diagnosis codes, procedures, laboratory tests, medications, and hospital admissions^39^. For model validation using genetic data, samples linked to genetic information collected by the UCLA ATLAS Community Health Initiative (ATLAS)^39^ were used as a holdout set (refer to Validation of Holdout Set Predictions Using Polygenic Risk Scores and *APOE-ε4*). ATLAS biological samples were collected during routine clinical lab work performed at a UCLA Health laboratory and then genotyped using a customized Illumina Global Screening Array. Participants watched a short video explaining the initiative’s goals and had their consent recorded. For training and testing, non-ATLAS patients with selected EHR features were used. As the EHR and genetic data were de-identified, this study was exempt from human subject research regulations (UCLA IRB# 25-1232).

### Sampling of positive (AD) and unlabeled data

We included patients with non-missing demographics (age, sex, self-reported race and ethnicity in the NH-white, NH-AfAm, HL, or EA group). We further included patients using the following criteria: 1) patients must have an average encounter of at least 1 per year and record length of at least 5 years to ensure regular health system visits; and 2) patients must be between the ages of 65 and 90 during their last visit.

From the records of eligible patients, we removed comorbidities represented by International Classification of Diseases (ICD)-10 diagnosis codes that are not risk factors of AD. We excluded diagnoses in chapters XV (Pregnancy, childbirth and the puerperium), XVI (Certain conditions originating in the perinatal period), and XVII (Congenital malformations, deformations and chromosomal abnormalities). We also excluded diagnoses in XX (External causes of morbidity and mortality), except those beginning with “W” as they include slipping, tripping, stumbling and falls, which are known AD comorbidities. In addition, we excluded F02.80 (Dementia in other diseases classified elsewhere without behavioral disturbance) because it is a concurrent diagnosis code with G30 (AD)^68^. We then mapped the ICD-10 codes with maximum granularity to phecodes, manually curated groups of ICD codes intended to capture clinically meaningful concepts, using Phecode Map 1.2 with ICD-10-CM codes^69^. This mapping reduced the dimensionality of our data and avoided redundant information from ICD codes within the same group, which may be highly correlated. Only the first encounter for each phecode was retained. After performing patient and record-level filtering, positive and unlabeled samples were selected based on presence and absence, respectively, of at least one diagnosis of AD, indicated by G30. We performed 1000 random 80/10/10 stratified train/validation/test splits by labeled AD status on non-ATLAS patients (*N* = 97,403). Kruskal-Wallis test followed by Games-Howell or Pearson’s chi-squared test were performed to determine statistical significance among the distributions. ATLAS patients (*N* = 18,305) were held out for model validation using genetic data.

### Selection of enriched diagnoses in AD patients

To select important phecodes for modeling, we fit a logistic regression model [parglm (v. 0.1.7)^70^] with AD as a binary outcome variable on training data for each of the 1638 unique phecodes, excluding 290.11 (AD), over 1000 random splits. Unlabeled patients were treated as controls, although they contain some positive cases. The goal of feature selection was to identify phecodes most strongly associated with AD in an unbiased manner for subsequent steps in the PUL framework. Assigning negative labels from the onset based on the absence of proxy AD ICD codes or medications was avoided as it would not align with the goal of our PUL framework. Logistic regression was chosen as the feature selection method for computational efficiency and to control for covariates. We performed min-max scaling on patient age since last visit, record length, record density, number of encounters, and number of diagnoses, and adjusted for them in our models along with sex and self-reported race and ethnicity. Phecodes of high risk for AD were determined based on statistical significance (*p* < 0.05, Wald test) following Bonferroni correction (0.05/1,638), and a prevalence of at least 1% in all patients. Across all training sets from the 1000 splits, a total of 458 unique phecodes were identified as significant. The mean significant phecodes per training set was 280.

### Baseline models

In this study, all models are classification models aimed at identifying undiagnosed AD cases using proxy ICD codes and medications. Here, “prediction” refers to classifying potential existing cases, not forecasting future development of AD.

As baselines for comparison, we tested 2 models, both of which were trained on noisy negative labels (i.e., labeling all unlabeled patients as negative). Although it is obviously incorrect to assume that all unlabeled patients are negative, this assumption is popular in practice as it enables the direct application of supervised binary classification^31,54^. Further, training on positive and noisy unlabeled data is reasonable because the class posterior (the probability that an instance belongs to the positive class) for classifiers trained on positive and negative labeled data is monotonically related to the posterior for classifiers trained on positive and unlabeled data^71^. In other words, as the class prior probability increases for the positive vs. negative classifier, it also increases for the positive vs. unlabeled classifier (though not necessarily linearly). The 2 baseline models are as follows: 1) a supervised model trained using only demographics and a list of manually curated AD risk factors supported by expert domain knowledge and evidence from the literature [Supervised (risk factors)]^14^; 2) a supervised model trained using all significant features from feature selection (refer to Selection of Enriched Diagnoses in AD Patients) [Supervised (full)]. We included the following AD risk factor phecodes in model 1: 249 (Secondary diabetes mellitus), 250.2 (Type 2 diabetes), 250.22 (Type 2 diabetes with renal manifestations), 250.24 (Type 2 diabetes with neurological manifestations), 250.25 (Diabetes type 2 with peripheral circulatory disorders), 272.1 (Hyperlipidemia), 401 (Hypertension), 401.1 (Essential hypertension). To obtain final predictions for each model, we selected the probability cutoff that maximizes the Matthew’s Correlation Coefficient (MCC) for unlabeled data in the validation set and applied it to the test set.

### Semi-supervised positive unlabeled learning with pre- and post-processing racial bias mitigation

We developed a 4-step positive unlabeled learning (PUL) framework to obtain AD predictions for unlabeled patients in the test set, which we refer to as semi-supervised positive unlabeled learning (SSPUL) (Fig. 2a, b). To enable learning from positive and unlabeled data, we assumed that labeled AD cases are selected at random and there exists a probabilistic gap such that positive instances which resemble negative instances more are less likely to be labeled^31^. The selected at random assumption is a weaker variant of the selected completely at random assumption, which states that labeled instances are an i.i.d. sample of the positive distribution^31^. The selected at random assumption states that labeled instances are a biased sample from the positive distribution^31^. This assumption of the labeling mechanism is reasonable because there are a multitude of reasons for why some AD patients are diagnosed while others are not. Examples include providers’ assumption that cognitive changes are normal rather than pathologic, providers not following up on the symptoms and complaints of AD patients from underrepresented groups, and socioeconomically disadvantaged AD patients’ lack of access to healthcare^6,53^. Therefore, NH-white patients with high socioeconomic status and access to unbiased providers may be more likely to be diagnosed (i.e., be a labeled positive instance).

We implemented our PUL framework by first training a model to classify LP and unlabeled patients using a GLM classifier^31,72^. To identify reliable negatives (RNs) from unlabeled patients, we calculated the probabilistic gap for each patient, defined as the difference between the probability of an instance having a label given its features and the complement: ΔPr(x) = Pr(y = 1|x) – Pr(y = 0|x)^31^. RNs were determined based on the following criteria: 1) probabilistic gap is *smaller than* the smallest observed probabilistic gap of the LPs (ΔP_LP_); 2) age during last visit is at least 70 years; 3) does not have any phecode falling in the AD phecode exclusion range (290–292.99). To minimize potential bias that may be introduced by extreme values (i.e., bias resulting from the smallest probabilistic gap being an outlier), we randomly sampled the probabilistic gaps of 500 LPs 1000 times, then took the mean of the minimums. RNs were manually reviewed by a domain expert for validation (refer to Validation of Reliable Negatives).

For the second step, we assigned positive and negative labels for a subset of the remaining unlabeled patients using race and ethnic-specific probabilistic criteria in order to increase the size of our training set while mitigating bias that may arise from overrepresentation of patients belonging to a particular race and ethnicity prior to training (i.e., pre-processing bias mitigation). To do this, we first used LPs and RNs as input for training a separate classifier. After obtaining updated predicted probabilities for LPs and RNs from the trained classifier, we subset the training data by race and ethnicity to obtain race-specific ΔP_LP_ and race-specific observed probabilistic gaps of RNs (ΔP_RN_). For each race and ethnicity subset, additional positives (APs) were determined based on having a probabilistic gap that is *greater than* the smallest ΔP_LP_, while additional negatives (ANs) were determined based on having a probabilistic gap that is *smaller than* the largest ΔP_RN_. Among all APs satisfying the probabilistic gap criterion, we selected those with the largest probabilistic gap such that the prevalence of total positive labels (LP and AP) for each race and ethnicity matches U.S. Census-adjusted AD population prevalence estimates from the Chicago Health and Aging Project (10% for NH-white, 18.6% for NH-AfAm, and 14% for HL)^11^ and meta-analysis from previous work (7.4% for EA)^12^.

For the third step, we used all labeled (LPs) and pseudo-labeled (RNs, APs, ANs) patients as input for training a final classifier, to which we applied the test set. In the last step, we performed post-processing bias mitigation by selecting the probability cutoff that optimizes the group benefit opportunity (GBE)^38^ for each race and ethnicity (refer to Evaluation Metrics) for unlabeled data in the validation set and applied it to the test set. For GBE, the expected prevalence was calculated from AD ICD codes, and proxy AD ICD codes and medications for a given racial and ethnic group. We implemented the Nelder-Mead optimization algorithm^73^ using the optim function in R such that the error from the target GBE of 1 is minimized. We compare the discrimination performance and fairness of baseline models and vanilla 2-step PUL (classifier 2) with SSPUL (final classifier) and report statistical significance using Wilcoxon signed rank test with Bonferroni correction in Table 2, Supplementary Table 2, and Supplementary Fig. 3. To investigate the impact of GBE optimization on discrimination performance and fairness, we also report discrimination performance and fairness metrics using race-specific optimal GBE cutoffs or the MCC cutoff for the baseline models and SSPUL, respectively (Supplementary Tables 4–6).

### Classifier selection

To select the best classifier for each model, we used the AutoML feature from the H2O package^74^ (v. 3.44.0.3) in R (v. 4.2.2) to train and tune 4 classifiers: Generalized Linear Model (GLM), Gradient Boosting Machine (GBM), Extreme Gradient Boosting (XGBoost), and Distributed Random Forest (DRF). Oversampling of patients in the minority class was implemented prior to training to address class imbalance. For both baseline models and steps 1 and 2 of SSPUL, the minority class was LPs. For step 3 of SSPUL, the minority class was LPs + additional (pseudo-labeled) positives. To reduce training time over 1000 splits, we applied AutoML across 10 initial splits, then selected the classifier to be applied for the remaining 990 splits based on the best mean area under the precision recall curve (AUCPR) (Supplementary Table 8). We selected GLM as the classifier for the baseline models and step 1 of SSPUL, and DRF as the classifier for step 2 of SSPUL as they achieved the best mean AUCPR, evaluated on 10 initial training sets. A higher AUCPR indicates better separability of LPs from unlabeled patients in step 1 and from RNs in step 2, reflected in more confident predicted probabilities. This improves the quality of pseudo-labels by minimizing false positives and false negatives. For step 3 of SSPUL, we selected XGBoost as the classifier as it achieved the best mean AUCPR, evaluated on 10 initial validation sets.

### Validation of reliable negatives

One domain expert (TSC) manually reviewed the diagnoses and medications of 100 random RNs. No RN patient was prescribed a proxy medication for dementia. No RN patient had G chapter (diseases of the nervous systems) or R40-46 (symptoms and signs involving cognition, perception, emotional state and behavior) ICD codes that suggested AD or dementia, except for one RN with memory loss (R41.3). Common G or R40-46 codes among RNs were dizziness, cerebrovascular disease, or tremor. A few RN were diagnosed with encephalopathy.

### Validation of test set predictions using proxy AD ICDs and medications

We validated final prediction labels of the test set using proxy AD diagnosis measures, which included alternative diagnosis ICD codes and medications for dementia. These can be considered silver standards and have been used previously to evaluate unlabeled cases predicted by semi-supervised learning^65^. AD proxy ICD codes included: vascular dementia (F01, F01.5, F01.50, F01.51, F01.0, F01.1, F01.2, F01.3, F01.9, F01.511, F01.518); dementias with cerebral degenerations (G31.0, G31.01, G31.09, G31.1, G31.83); memory loss (R41.1, R41.2, R41.3); mild cognitive impairment (G31.84); unspecified dementias (F03.9, F03.90, F03.91, F03.911, F03.918, F02, F02.8, F02.80, F02.81, F02.0, F02.811, F02.818); senile dementia (F03); and other cerebral degenerations (G31.85). Medications included donepezil, rivastigmine, galantamine, memantine, memantine/donepezil combination, aducanumab, and lecanemab. Unlabeled patients without any proxies were considered healthy controls.

### Validation of holdout set predictions using polygenic risk scores and *APOE-ε4*

Polygenic risk scores (PRS) estimate the heritable risk of an individual for developing a particular disease by combining information from many SNPs associated with the disease or trait of interest in genome-wide association studies (GWAS)^75^. As another method for validation, we generated PRS using genotype data from ATLAS patients in a holdout set (*N* = 18,305). We selected the late-onset AD GWAS conducted by Kunkle et al.^49^ for building PRS based on its large sample size (21,982 cases and 41,944 controls). We first performed quality control (QC) using PLINK v1.9^76^ following established guidelines^39^. Subsequently, genotype imputation was performed using the Michigan Imputation Server (Das) to enhance the coverage of genetic variants. We used the LDpred2 tool from the bigsnpr package (v. 1.6.1) to build our PRS^77^. This tool updates SNP weights based on linkage disequilibrium (LD) information from a reference population, which in our case was the European genetic ancestry sample from the 1000 Genomes Project^77,78^. We calculated the sum of a patient’s risk allele dosages, weighted by risk allele effect sizes, to obtain the final PRS. To compare the PRSs of LPs and predicted labels, we applied each final SSPUL classifier trained on one of 1000 random splits on the holdout set, measured the mean PRS of predicted positives or predicted negatives from each split, then aggregated them to obtain the mean of PRS means. LPs had a single PRS mean as they were independent of the model’s predictions. We stratified the mean of PRS means by self-reported race and ethnicity and compared them across classifications using Wilcoxon signed-rank test with Bonferroni correction.

In addition to PRS, we validated our model using apolipoprotein E (*APOE*) *ε4* allele count. *APOE-ε4* is a significant genetic risk factor for AD and is associated with an increase in the levels of amyloid deposition, which leads to AD onset^47,48^. We compared *APOE-ε4* allele count across classifications and stratified our analysis similarly to PRS validation.

### Evaluation metrics

We used the area under the receiver operating characteristic curve (AUC), sensitivity, specificity, and precision to compare model discrimination performance, stratified by race and ethnicity. To account for class imbalance, we also included balanced accuracy (BA) and AUCPR. Balanced Brier score was additionally included to compare model calibration performance, which is indicative of the quality of the predicted probabilities with respect to the proxy-validated class labels. A well-calibrated model will have higher predicted probabilities for proxy-validated AD cases than for controls and hence a lower balanced Brier score^40,41^. Unlike the traditional Brier score^79^, the Balanced Brier score is robust to class imbalance as it is composed of stratified Brier scores, each calculated separately over positive or negative samples (Eqs. 1–3)^41^. By measuring each model’s classification performance with regards to both its discrimination and calibration capabilities in a balanced fashion, we established a more accurate and comprehensive comparison. Of note, although these metrics are traditionally applied in supervised learning frameworks, they have been utilized in other PUL frameworks where proxies are used for validation^80,81^ or the ground truth labels are known^82,83^.

1 $${Brie}{r}^{+}=\frac{{\sum }_{{y}_{i}=1}{\left({y}_{i}-\hat{{y}_{i}}\right)}^{2}}{{N}_{{pos}}}$$

2 $${Brie}{r}^{-}=\frac{{\sum }_{{y}_{i}=0}{\left({y}_{i}-\hat{{y}_{i}}\right)}^{2}}{{N}_{{neg}}}$$

3 $${Balanced\; Brier}={Brie}{{r}}^{+}+{Brie}{{r}}^{-}$$

To compare model fairness, we assessed the differences between an unprivileged group (NH-AfAm, HL, or EA) and the privileged group (NH-white) in obtaining the favored outcome (AD) with respect to the following metrics: specificity, precision, BA, equal opportunity (EO), and group benefit equality (GBE) (Eq. 4). EO asserts that the sensitivities for two groups are equal (Eq. 5)^84^. Because the prevalence of AD differs among NH-white, NH-AfAm, HL, and EA groups, an even proportion of patients in each group identified to have AD is unlikely. For this reason, we also considered group benefit equality (GBE)^38^, which enforces that the rate at which AD is diagnosed based on ICD codes and predicted to occur within a race and ethnicity, and the rate at which it occurs in the EHR by virtue of AD ICD code diagnosis and proxy AD ICD codes and medication is equal. This relationship can be expressed as a ratio (Eq. 6), which is 1 in the ideal case, indicating that the number of AD predictions combined with LPs matches that of diagnosed and proxy-validated AD cases. By aggregating the absolute differences across unprivileged groups for each metric, we obtained metric-specific parity losses (Eq. 7), which were summed to yield the cumulative parity loss (Eq. 8)^42^.

4 $${{M}}_{{difference}}={{M}}_{{unprivileged}}-{{M}}_{{privileged}};{M}\in \{{specificity},\; {precision},\; {BA},\; {EO},\; {GBE}\}$$

5 $${EO}:{P}\left(\hat{{Y}}=1|{A}={unprivileged},{Y}=1\right)={P}\left(\hat{{Y}}=1|{A}={privileged},{Y}=1\right);{unprivileged}\in \{{\rm{NH-}}{\rm{AfAm}},\; {\rm{HL}},\; {\rm{EA}}\},\; {privileged}={\rm{NH-}}{\rm{white}}$$

6 $${GBE}:\frac{P\left(\hat{Y}=1|A=a\right)}{P\left(Y=1|A=a\right)}=1;{a}\in \{{\rm{NH-}}{\rm{White}},\; {\rm{NH-}}{\rm{AfAm}},\; {\rm{HL}},\; {\rm{EA}}\}$$

7 $${Parity}\;{loss}_{M}=\mathop{\sum}\limits_{unprivileged}|{{M}}_{{difference}}|$$

8 $${Cumulative}\; {parity}\; {loss}=\mathop{\sum }\limits_{{M}}{parity}\; {loss}_{{M}}$$

### Factor analysis of mixed data and selection of true positive subset

Taking the top 20 predictive features of the final classifier by scaled importance, we performed factor analysis of mixed data (FAMD) to visualize the final classifications of the test set of one split in a 2-dimensional Euclidean feature space. FAMD is a principal component method that enables dimensionality reduction of both continuous (e.g., age during last visit) and categorical variables (e.g., phecodes) by scaling the former to unit variance and crisp coding, followed by scaling the latter using the specific scaling of multiple correspondence analysis^43^. FAMD was performed using the FactoMineR package (v. 2.11)^43^ in R (v. 4.2.2).

One of the potential benefits of PUL is predicting true positives (TPs) (i.e., proxy-validated predicted positives) with a feature space that differs than that of LPs, potentially revealing novel informative features. We identified this TP subset in our data by selecting predicted positives that did not overlap with LPs (except one outlier) in a 2-dimensional Euclidean feature space visualized via FAMD, and subsequently filtering for TPs. To better understand the relationship between the TP subset, LPs, and top 20 features, we plotted the coordinates of their projections onto the same feature space. To do this, we re-ran FAMD after discretizing top continuous features, then obtained the coordinates of the projections on the categories of each feature (*N* = 15 top phecodes * 2 categories + 5 top continuous features (age during last visit, number of encounters, number of diagnoses, record length, record density) * 4 categories = 50 total categories) on the feature space from the first FAMD run. We then performed 2 proportion z-test to compare the prevalence of features with coordinates that are closer to those of the TP subset relative to LPs and vice versa.

### SHAP analysis

Originating from cooperative game theory, SHapley Additive exPlanations (SHAP) analysis is widely used for explaining machine learning models by assigning to each feature an importance value that represents its contribution to the model’s output^45^. SHAP values are determined by calculating the marginal contribution of each feature to the final model prediction with respect to all possible subsets of features. To assess the contribution of each TP subset-specific feature to the final predictions of the TP subset and LP sample described in Factor Analysis of Mixed Data and Selection of True Positive Subset, we compared the mean absolute SHAP values of each feature for the 2 groups using Mann-Whitney U test with Bonferroni correction. For this analysis, we implemented Tree SHAP^85^, a variant of SHAP that efficiently computes exact Shapley values for tree-based models, using H2O’s predict_contributions function. As input, we included all significant features from feature selection.

We investigated potential disparities in feature importance across racial and ethnic groups by examining differences in the magnitude and direction of SHAP values. To do this, we sampled 100 unlabeled patients of each race and ethnicity from a random test set and evaluated how feature contributions differed between NH-white and unprivileged groups. We then quantified the magnitude of feature influence by averaging the absolute SHAP values of each top feature within each racial and ethnic group. To determine differences in the direction of SHAP values, we analyzed the correlation between each feature’s value and its corresponding SHAP value separately for each group using the Pearson correlation coefficient. We tested the null hypothesis that there is no difference in the correlation between feature values and SHAP values across NH-white and unprivileged groups. To simulate the null distribution of correlation differences, we randomly shuffled racial group labels (NH-white and NH-AfAm; NH-white and HL; NH-white and EA) 1000 times under the assumption that if correlation is independent of race and ethnicity, then permuting these labels should not yield any meaningful correlation difference. We then compared the observed correlation difference to the null distribution to assess whether it was more extreme than what would be expected by random chance. We defined the p-value as the proportion of correlation differences resulting from permutation that were equal to or more extreme than the observed correlation difference.

### Sensitivity analysis of self-reported race and ethnicity features

To investigate the impact of self-reported races and ethnicities on differential sensitivity performance across models, we conducted a sensitivity analysis. In this analysis, we recoded each race and ethnicity as another race and ethnicity (e.g., NH-white to NH-AfAm) while keeping all other features fixed in the test set, obtained predicted probabilities via the final XGBoost classifier, and used the original classification cutoff to acquire updated sensitives. For example, for models that use GBE cutoffs, the optimal GBE cutoff for NH-whites prior to recoding would be applied to NH-whites that are recoded to NH-AfAms. If the model were racially unbiased, then the predicted probability of a patient having AD, given the same set of features except race and ethnicity, should be the same or similar. Therefore, applying the GBE cutoff before and after recoding should yield a similar set of predicted labels. After recoding, we compared across models the gain or loss in sensitivity for each original race and ethnicity.

### Sensitivity analysis of proxy label distribution shifts

The different proxy subsets used for testing model robustness to proxy label distribution shifts included: 1) subsets where each proxy ICD was removed one at a time, 2) subsets defined by grouping proxy ICDs into phecodes and removing one phecode at a time, 3) subsets removing 5 randomly selected ICDs at a time, and 4) a subset removing all proxy medications. After removing the ICD code(s) or medications for the respective subset, patients that still have proxies were validated as cases while those without proxies were validated as healthy controls. Based on each subset, we optimized race-specific GBE cutoffs and evaluated discrimination performance on 1000 random validation and test sets, respectively.

## Supplementary information

Supplementary Information

## Data Availability

Individual electronic health record data are not publicly available due to patient confidentiality and security concerns. Collaboration with the study authors who have been approved by UCLA Health for Institutional Review Board-qualified studies are possible and encouraged.

## Code availability

The underlying code and training/validation dataset for this study is not publicly available due to proprietary and privacy reasons. The coding framework can be made available to qualified researchers on reasonable request from the corresponding author.
