# Supplementary material for: Fair positive unlabeled learning for predicting undiagnosed Alzheimer’s disease in diverse electronic health records
Source: NPJ Digit Med. 2025 Nov 27;8:730. doi: 10.1038/s41746-025-02111-1 (PMC12661022; doi:10.1038/s41746-025-02111-1)
Supplement: Supplementary file 1 — Supplementary Information [file 41746_2025_2111_MOESM1_ESM.docx]

**Supplementary Figure 1: Confusion matrices for predictions.**

**
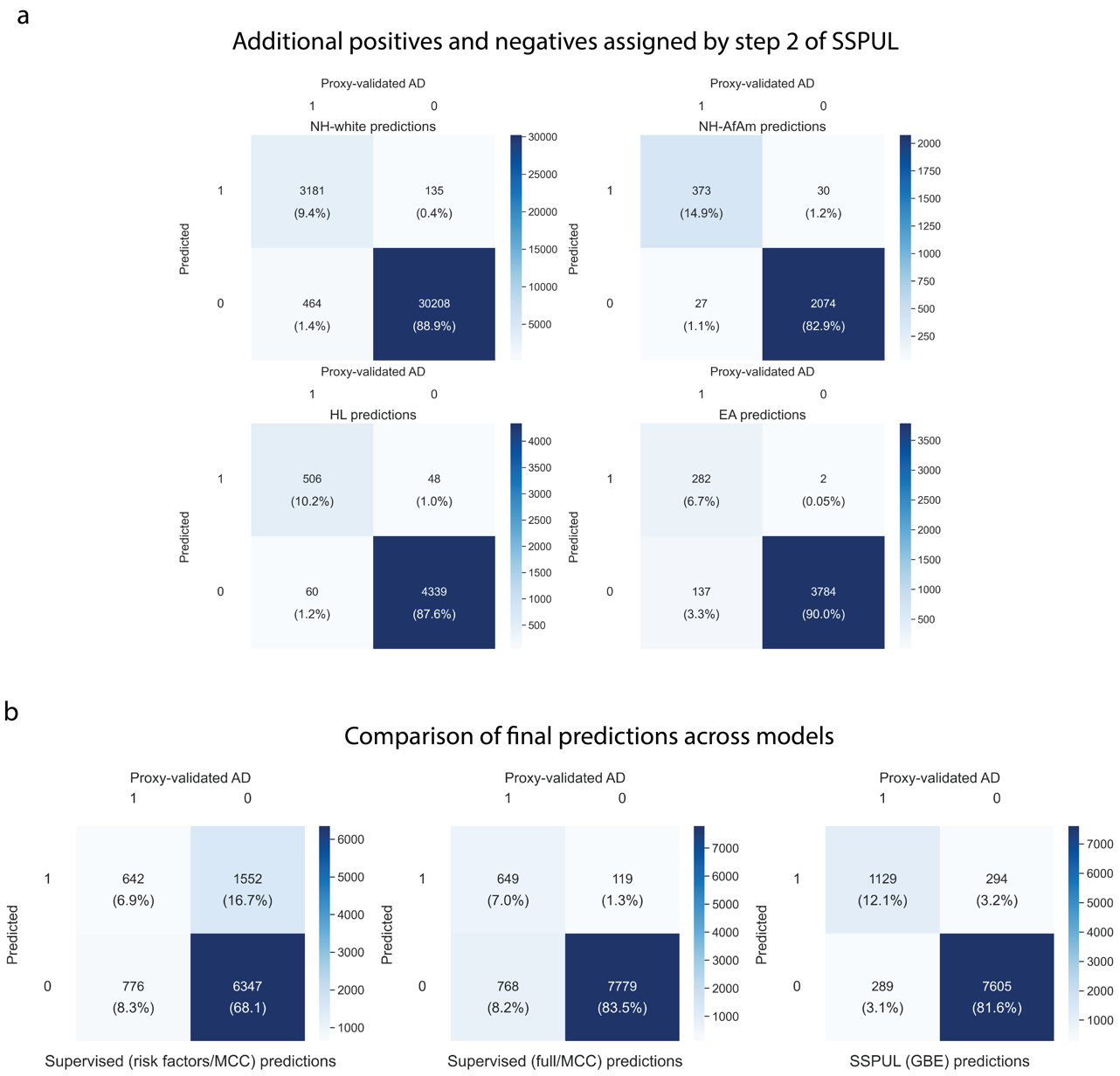
**

a) Additional positives and additional negatives assigned by step 2 of SSPUL. Values in confusion matrices are means of 1000 training sets. b) Comparison of final predictions across models. Values in confusion matrices are means of 1000 test sets. AD=Alzheimer’s disease, EA=East Asian, GBE=group benefit equality, HL=Hispanic Latino, MCC=Matthew’s Correlation Coefficient, NH-AfAm=non-Hispanic African American, NH-white=non-Hispanic white, SSPUL=semi-supervised positive unlabeled learning.

**Supplementary Figure 2: Comparison of calibration performance across models.**

**
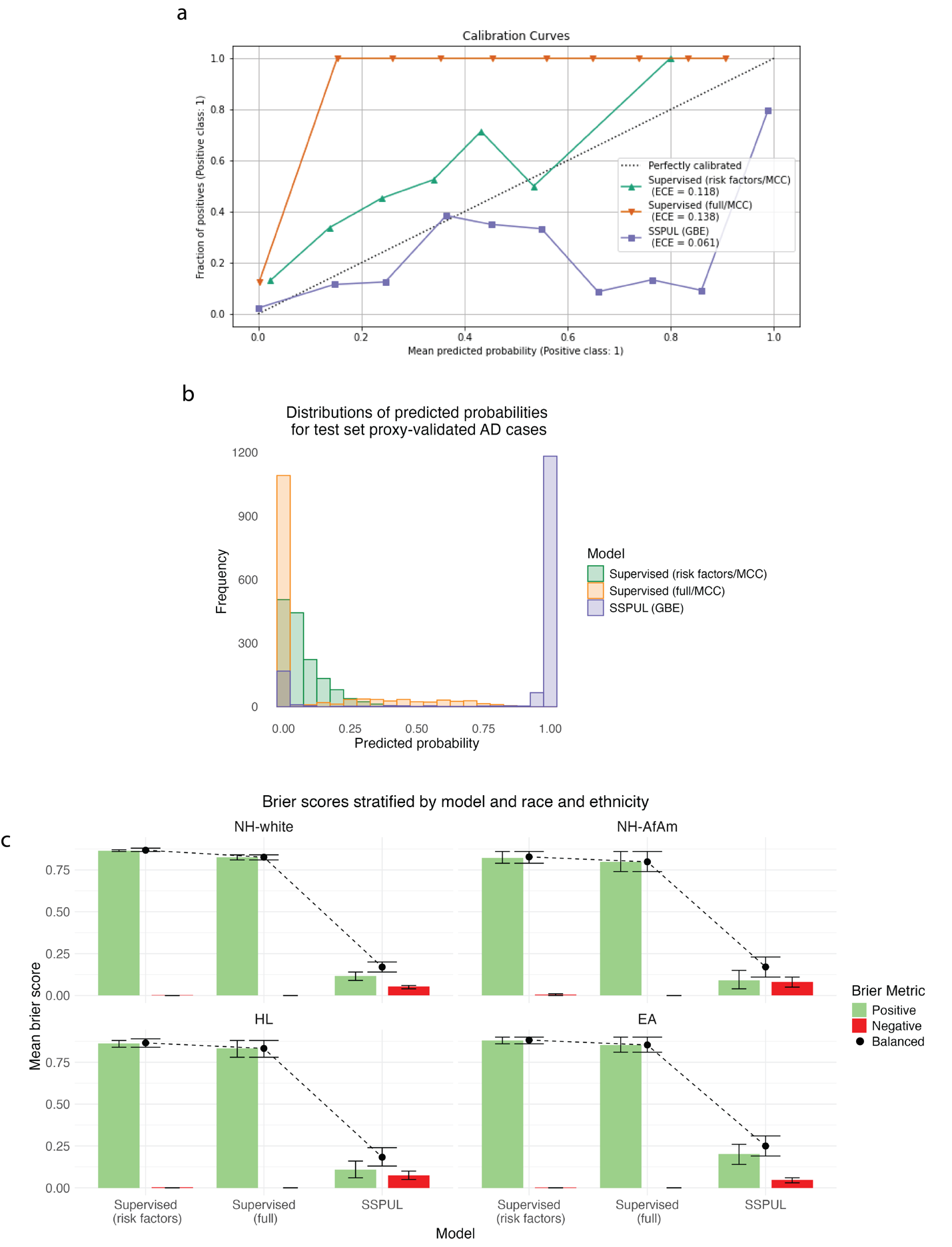
**

a) Calibration curves of SSPUL and baseline models. Each bin represents approximately 10% mean predicted probability. Supervised (risk factors) had a maximum predicted probability of 0.79, resulting in missing points in bins 8-10 from the corresponding calibration curve. b) Distributions of predicted probabilities for test set proxy-validated AD cases, stratified by model. c) Brier scores stratified by model and race and ethnicity. EA=East Asian, ECE=Expected Calibration Error, GBE=group benefit equality, HL=Hispanic Latino, MCC=Matthew’s Correlation Coefficient, NH-AfAm=non-Hispanic African American, NH-white=non-Hispanic white, SSPUL=semi-supervised positive unlabeled learning.

**Supplementary Figure 3: Comparing fairness by cutoff method.**

**
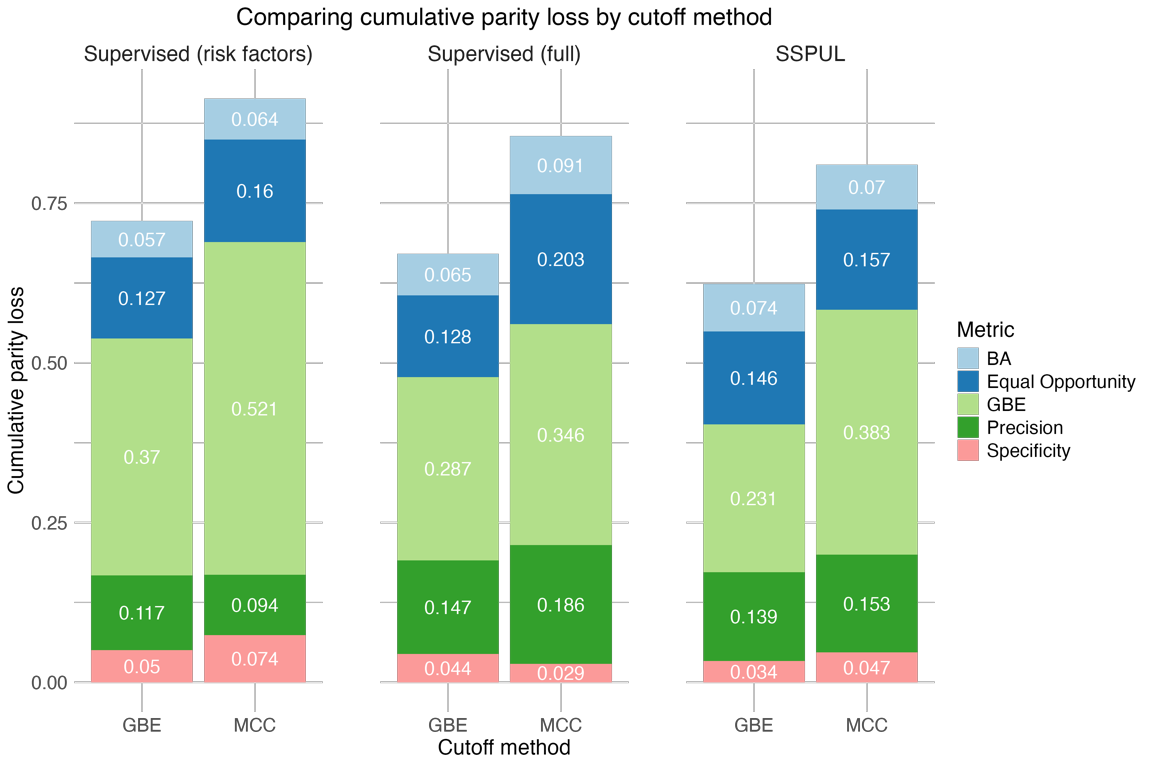
**

Fairness metrics were averaged over 1000 test sets. Cutoffs for MCC-maximized models were selected by maximizing the MCC for unlabeled data in the validation set using proxy labels. Cutoff for GBE-optimized models were selected by optimizing the GBE for each race/ethnicity in the validation set. A lower cumulative parity loss reflects better overall fairness. BA=balanced accuracy, GBE=group benefit equality, MCC=Matthew’s Correlation Coefficient, SSPUL=semi-supervised positive unlabeled learning.

**Supplementary Figure 4: Comparison of SHAP value magnitude and direction among the top 20 features across racial and ethnic groups.**

**
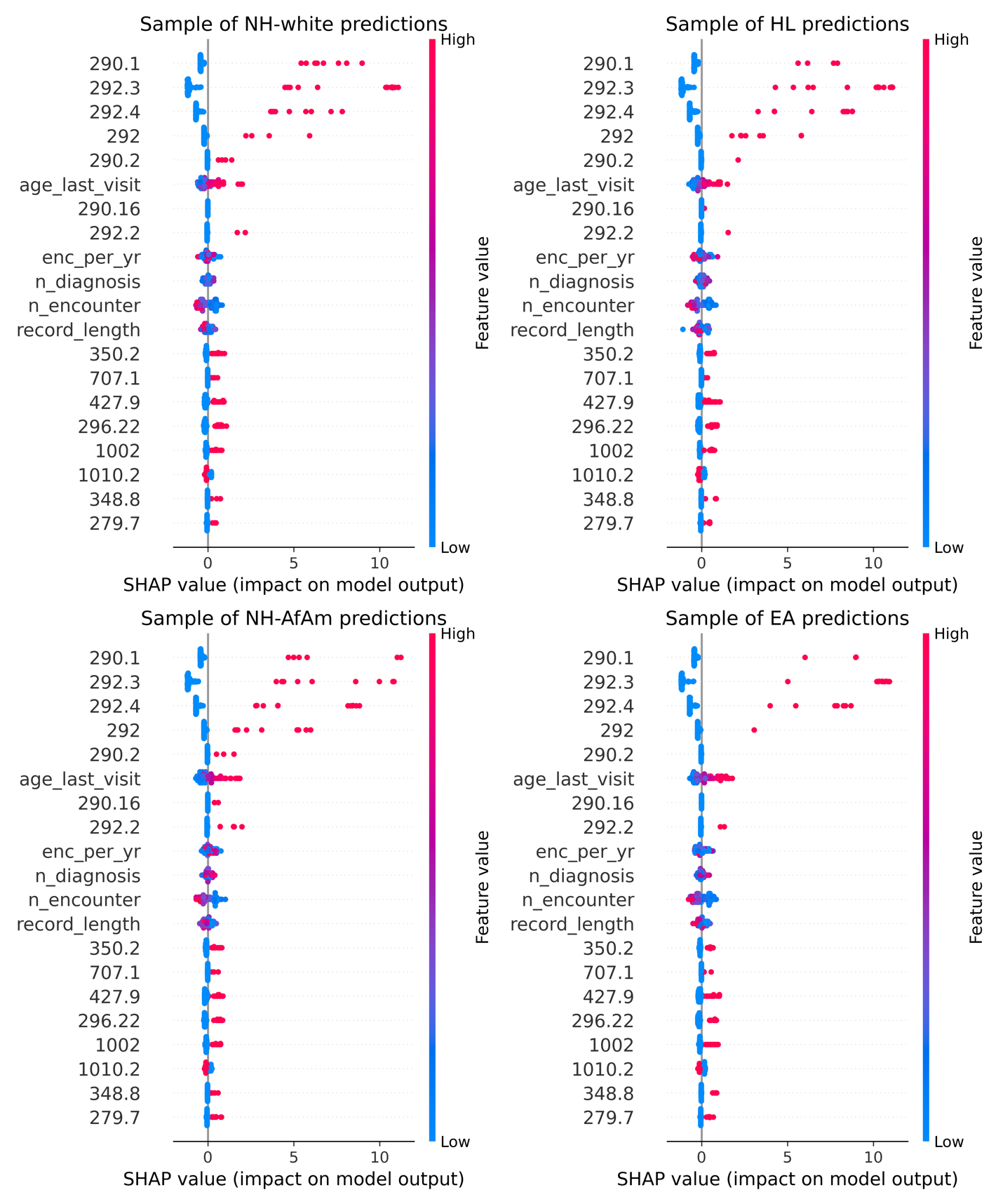
**

Feature values for phecode features are binary (0 = low/blue, 1 = high/red). Feature values for continuous features (age at last visit, record density (per year), number of diagnoses, number of encounters, and record length) were min-max scaled. Purple represents intermediate values for continuous features. EA=East Asian, HL=Hispanic Latino, NH-AfAm=non-Hispanic African American, NH-white=non-Hispanic white, SHAP=SHapley Additive exPlanations.

**Supplementary Figure 5: AD genetic risk association with classification and proxy-validated labels.**

**
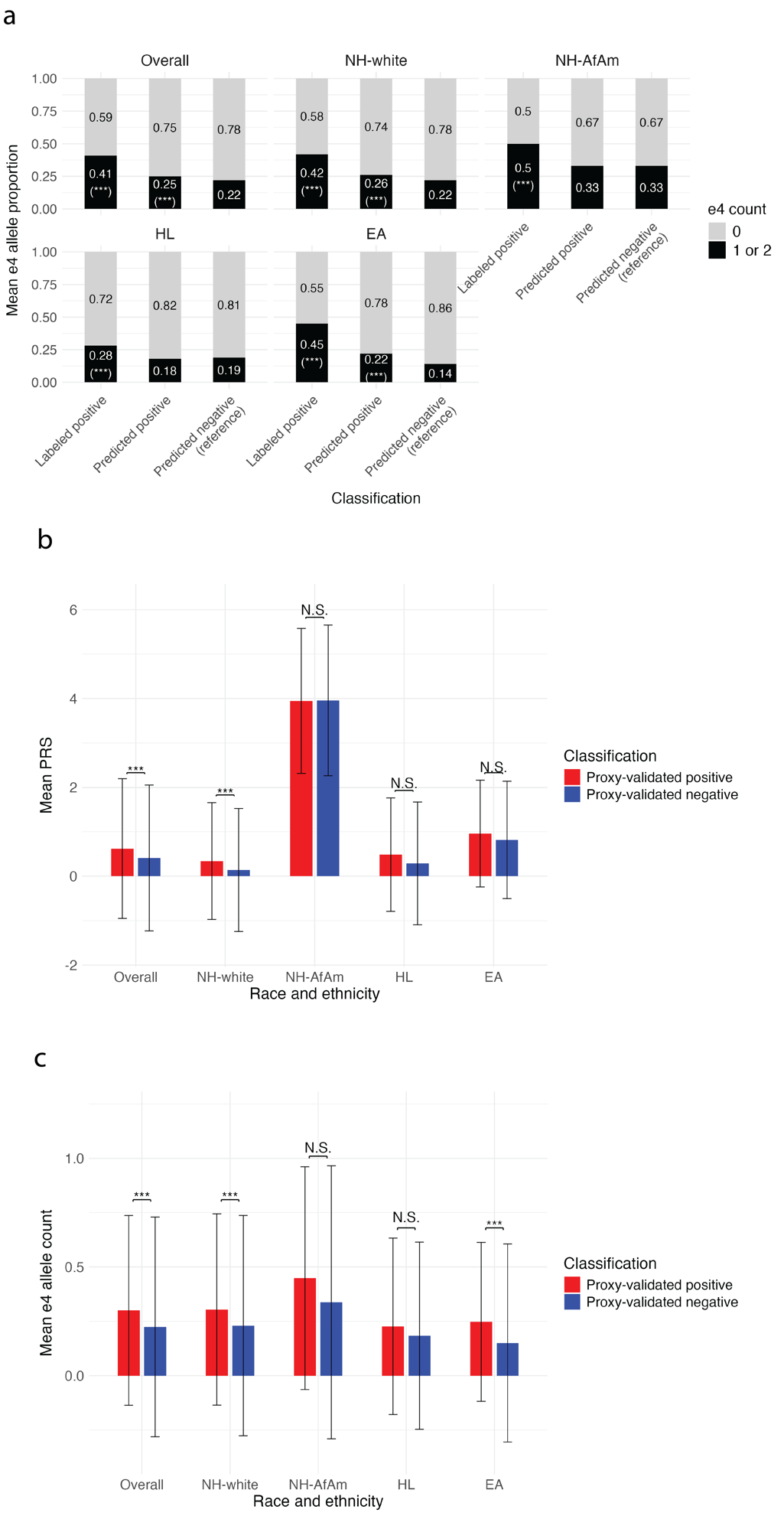
**

a) ε4 allele count proportions of holdout set prediction labels. b) PRS and ε4 allele counts of proxy-validated labels. Prediction labels for the holdout set were inferred using 1000 trained models. c) The ε4 allele proportions for labeled positives and each prediction label were obtained for each iteration, then averaged. EA=East Asian, HL=Hispanic Latino, NH-AfAm=non-Hispanic African American, NH-white=non-Hispanic white, N.S.=not significant (p > 0.05), PRS=polygenic risk score.

| **Supplementary Table 1: Distributions of final labels of test set, stratified by sex and self-reported race and ethnicity** | | | | | | |
| --- | --- | --- | --- | --- | --- | --- |
|  | Labeled AD | Predicted positive | Predicted negative | Labeled AD prevalence (%) | SSPUL predicted prevalence (%) | Labeled and proxy-validated AD prevalence (%) |
| Race and ethnicity | |  |  |  |  |  |
| NH-white | 306  (288, 324) | 1023  (954, 1089) | 5760  (5656, 5852) | 4.3  (4.1, 4.6) | 18.7  (17.8, 19.7) | 18.7  (17.9, 19.6) |
| NH-AfAm | 35  (25, 46) | 97  (73, 130) | 468  (426, 510) | 5.8  (4.2, 7.7) | 22.0  (18.1, 27.7) | 21.5  (18.6, 24.6) |
| HL | 47  (35, 58) | 152  (124, 187) | 880  (821, 938) | 4.3  (3.3, 5.5) | 18.5  (15.6, 21.5) | 18.3  (16.2, 20.6) |
| EA | 38  (27, 49) | 150  (126, 177) | 785  (735, 834) | 3.9  (2.8, 5.0) | 19.3  (16.7, 22.1) | 19.4  (17.3, 21.6) |
| Sex |  |  |  |  |  |  |
| Male | 150  (131, 167) | 615  (564, 670) | 3375  (3285, 3469) | 3.6  (3.2, 4.0) | 18.5  (17.2, 19.9) | 17.8  (16.8, 18.8) |
| Female | 275  (258, 294) | 808  (747, 872) | 4518  (4420, 4616) | 4.9  (4.6, 5.2) | 19.3  (18.2, 20.4) | 19.7  (18.9, 20.7) |
| Total | 425 | 1423  (1345, 1507) | 7893  (7809, 7970) | 4.4 | 19.0  (18.2, 19.8) | 18.9  (18.2, 19.6) |
| Final labels reported are means of 1000 random test sets with 95% CI. AD=Alzheimer's Disease, HL=Hispanic Latino, NH-AfAm=non-Hispanic African American, NH-white=non-Hispanic white, SSPUL=semi-supervised positive unlabeled learning. | | | | | | |

| **Supplementary Table 2: Test set performance of models with modified cutoffs and vanilla 2-step PUL** | | | | | |  |
| --- | --- | --- | --- | --- | --- | --- |
|  |  |  |  |  |  |  |
| Race/ethnicity | Model | Sensitivity | Precision | Specificity | B. Accuracy |  |
| NH-white | Supervised (risk factors/GBE) | 0.33 | 0.33 | 0.88 | 0.61 |  |
|  |  | (0.30, 0.37) | (0.31, 0.36) | (0.87, 0.89) | (0.59, 0.62) |  |
|  | Supervised (full/GBE) | 0.6 | 0.6 | 0.93 | 0.77 |  |
|  |  | (0.57, 0.64) | (0.56, 0.64) | (0.92, 0.94) | (0.75, 0.78) |  |
|  | 2-step PUL (GBE) | 0.78 | 0.78 | 0.96 | 0.87 |  |
|  |  | (0.70, 0.85) | (0.70, 0.85) | (0.95, 0.97) | (0.82, 0.91) |  |
|  | SSPUL (MCC) | 0.85 | 0.8 | 0.96 | 0.9 |  |
|  |  | (0.80, 0.91) | (0.69, 0.93) | (0.93, 0.99) | (0.88, 0.92) |  |
|  | SSPUL (GBE) | 0.8 | 0.8 | 0.96 | 0.88 |  |
|  |  | (0.71, 0.87) | (0.71, 0.87) | (0.95, 0.98) | (0.83, 0.92) |  |
| NH-AfAm | Supervised (risk factors/GBE) | 0.36 | 0.37 | 0.87 | 0.62 |  |
|  |  | (0.25, 0.48) | (0.28, 0.48) | (0.83, 0.91) | (0.57, 0.67) |  |
|  | Supervised (full/GBE) | 0.62 | 0.62 | 0.92 | 0.77 |  |
|  |  | (0.52, 0.72) | (0.50, 0.74) | (0.89, 0.96) | (0.72, 0.82) |  |
|  | 2-step PUL (GBE) | 0.76 | 0.78 | 0.96 | 0.86 |  |
|  |  | (0.59, 0.89) | (0.64, 0.90) | (0.92, 0.98) | (0.78, 0.92) |  |
|  | SSPUL (MCC) | 0.88 | 0.75 | 0.94 | 0.91 |  |
|  |  | (0.79, 0.95) | (0.61, 0.93) | (0.89, 0.99) | (0.87, 0.94) |  |
|  | SSPUL (GBE) | 0.81 | 0.79 | 0.96 | 0.88 |  |
|  |  | (0.64, 0.92) | (0.63, 0.91) | (0.91, 0.98) | (0.79, 0.94) |  |
| HL | Supervised (risk factors/GBE) | 0.33 | 0.33 | 0.88 | 0.61 |  |
|  |  | (0.24, 0.41) | (0.25, 0.40) | (0.85, 0.92) | (0.57, 0.64) |  |
|  | Supervised (full/GBE) | 0.6 | 0.61 | 0.93 | 0.77 |  |
|  |  | (0.52, 0.68) | (0.51, 0.71) | (0.91, 0.96) | (0.73, 0.81) |  |
|  | 2-step PUL (GBE) | 0.74 | 0.75 | 0.96 | 0.85 |  |
|  |  | (0.57, 0.86) | (0.62, 0.87) | (0.93, 0.98) | (0.76, 0.91) |  |
|  | SSPUL (MCC) | 0.86 | 0.73 | 0.94 | 0.9 |  |
|  |  | (0.79, 0.93) | (0.60, 0.92) | (0.90, 0.99) | (0.87, 0.93) |  |
|  | SSPUL (GBE) | 0.77 | 0.77 | 0.96 | 0.87 |  |
|  |  | (0.60, 0.89) | (0.61, 0.90) | (0.92, 0.98) | (0.77, 0.93) |  |
| EA | Supervised (risk factors/GBE) | 0.34 | 0.34 | 0.87 | 0.61 |  |
|  |  | (0.26, 0.43) | (0.27, 0.42) | (0.84, 0.91) | (0.57, 0.65) |  |
|  | Supervised (full/GBE) | 0.57 | 0.57 | 0.92 | 0.74 |  |
|  |  | (0.47, 0.65) | (0.48, 0.67) | (0.89, 0.95) | (0.70, 0.78) |  |
|  | 2-step PUL (GBE) | 0.77 | 0.76 | 0.95 | 0.86 |  |
|  |  | (0.66, 0.85) | (0.68, 0.85) | (0.93, 0.97) | (0.80, 0.90) |  |
|  | SSPUL (MCC) | 0.76 | 0.82 | 0.97 | 0.86 |  |
|  |  | (0.66, 0.84) | (0.69, 0.96) | (0.93, 0.99) | (0.82, 0.90) |  |
|  | SSPUL (GBE) | 0.77 | 0.77 | 0.96 | 0.86 |  |
|  |  | (0.67, 0.84) | (0.69, 0.86) | (0.94, 0.98) | (0.81, 0.90) |  |
|  |  |  |  |  |  |  |
| Metrics reported are means of 1000 random test sets with 95% CI. Cutoffs for MCC-optimized models were selected by maximizing the MCC for unlabeled data in the validation set using proxy labels. Cutoff for GBE-optimized models were selected by optimizing the GBE for each race/ethnicity in the validation set. SSPUL (GBE) is shown for reference. B. Accuracy=balanced accuracy, EA=East Asian, GBE=group benefit equality, HL=Hispanic Latino, MCC=Matthew's correlation coefficient, NH-AfAm=non-Hispanic African American, NH-white=non-Hispanic white, PUL=positive unlabeled learning, SSPUL=semi-supervised positive unlabeled learning. | | | | | |  |

| **Supplementary Table 3: Changes in sensitivity across models after recoding self-reported race and ethnicity features** | | | | |
| --- | --- | --- | --- | --- |
|  |  |  |  |  |
| Race/ethnicity | Recoded race/ethnicity | Supervised (risk factors/MCC) | Supervised (full/MCC) | SSPUL (GBE) |
|  |  |  |  |  |
| NH-white | NH-AfAm | +0.07  (+0.05, +0.09) | +0.05  (+0.01, +0.09) | +0.01  (-0.03, +0.09) |
|  | HL | +0.01  (0.00, +0.03) | +0.07  (+0.04, +0.11) | +0.01  (-0.03, +0.09) |
|  | EA | -0.03  (-0.05, -0.01) | -0.02  (-0.05, +0.01) | +0.01  (-0.04, 0.06) |
| NH-AfAm | NH-white | -0.07  (-0.12, -0.02) | -0.04  (-0.10, 0.00) | -0.02  (-0.14, +0.03) |
|  | HL | -0.05  (-0.11, -0.01) | +0.02  (-0.02, +0.07) | 0.00  (-0.06, +0.05) |
|  | EA | -0.10  (-0.17, -0.04) | -0.06  (-0.12, -0.01) | -0.01  (-0.08, 0.00) |
| HL | NH-white | -0.01  (-0.04, 0.00) | -0.07  (-0.12, -0.03) | -0.03  (-0.16, +0.04) |
|  | NH-AfAm | +0.05  (+0.02, +0.10) | -0.02  (-0.07, +0.02) | -0.01  (-0.09, +0.07) |
|  | EA | -0.04  (-0.08, -0.01) | -0.09 (-0.15, -0.04) | -0.01  (-0.11, 0.00) |
| EA | NH-white | +0.03  (+0.01, +0.06) | +0.02  (-0.01, +0.06) | -0.01  (-0.06, +0.01) |
|  | NH-AfAm | +0.10  (+0.05, +0.16) | +0.07  (+0.02, +0.14) | 0.00  (0.00, +0.02) |
|  | HL | +0.04  (+0.01, +0.08) | +0.09  (+0.04, +0.16) | 0.00  (0.00, +0.03) |
|  |  |  |  |  |
| Mean changes in sensitivity from 1000 random test sets are reported with 95% CI. Cutoffs for supervised models were selected by maximizing the MCC for unlabeled data in the validation set using proxy labels. Cutoff for SSPUL model was selected by optimizing the GBE for each race/ethnicity in the validation set. The same cutoffs were applied after recoding. EA=East Asian, GBE=group benefit equality, HL=Hispanic Latino, MCC=Matthew's correlation coefficient, NH-AfAm=non-Hispanic African American, NH-white=non-Hispanic white, SSPUL=semi-supervied positive unlabeled learning. | | | | |

| **Supplementary Table 4: Comparison of test set fairness with respect to NH-AfAm and NH-white using MCC or GBE cutoffs** | | | | | | | |  |
| --- | --- | --- | --- | --- | --- | --- | --- | --- |
|  |  |  |  |  |  |  |  |  |
| Metric | Supervised (risk factors/MCC) | Supervised (risk factors/GBE) | Supervised (full/MCC) | Supervised (full/GBE) | 2-step PUL | SSPUL (MCC) | SSPUL (GBE) |  |
|  |  |  |  |  | (GBE) |  |  |  |
| BA | 0.02 | 0.01 | 0.03 | 0.01 | -0.01 | 0.01 | 0 |  |
|  | (-0.03, 0.08) | (-0.04, 0.07) | (-0.03, 0.08) | (-0.04, 0.06) | (-0.08, 0.04) | (-0.03, 0.04) | (-0.06, 0.04) |  |
| EO | 0.08 | 0.03 | 0.06 | 0.02 | -0.02 | 0.03 | 0 |  |
|  | (-0.02, 0.19) | (-0.09, 0.15) | (-0.04, 0.18) | (-0.08, 0.13) | (-0.18, 0.09) | (-0.05, 0.11) | (-0.12, 0.12) |  |
| Precision | 0.02 | 0.05 | -0.03 | 0.02 | 0 | -0.05 | -0.01 |  |
|  | (-0.06, 0.10) | (-0.06, 0.14) | (-0.14, 0.07) | (-0.10, 0.14) | (-0.12, 0.10) | (-0.14, 0.04) | (-0.13, 0.08) |  |
| Specificity | -0.04 | -0.01 | -0.01 | -0.01 | -0.01 | -0.02 | -0.01 |  |
|  | (-0.08, 0.00) | (-0.05, 0.03) | (-0.03, 0.00) | (-0.05, 0.03) | (-0.04, 0.02) | (-0.05, 0.00) | (-0.05, 0.01) |  |
| GBE | 0.16 | 0 | 0.1 | 0 | -0.02 | 0.12 | 0.03 |  |
|  | (-0.21, 0.56) | (-0.30, 0.35) | (-0.05, 0.26) | (-0.25, 0.27) | (-0.28, 0.22) | (-0.04, 0.31) | (-0.17, 0.31) |  |
|  |  |  |  |  |  |  |  |  |
| Metrics reported are means of differences between NH-AfAm and NH-white for 1000 random test sets with 95% CI. Cutoffs for MCC-optimized models were selected by maximizing the MCC for unlabeled data in the validation set using proxy labels. Cutoff for GBE-optimized models were selected by optimizing the GBE for each race/ethnicity in the validation set. BA=balanced accuracy, EO=equal opportunity, GBE=group benefit equality, MCC=Matthew's correlation coefficient, NH-AfAm=non-Hispanic African American, NH-white=non-Hispanic white, NPV=negative predictive value, PUL=positive unlabeled learning, SSPUL=semi-supervised positive unlabeled learning. | | | | | | | |  |

| **Supplementary Table 5: Comparison of test set fairness with respect to HL and NH-white using MCC or GBE cutoffs** | | | | | | |  |  |
| --- | --- | --- | --- | --- | --- | --- | --- | --- |
|  |  |  |  |  |  |  |  |  |
| Metric | Supervised (risk factors/MCC) | Supervised (risk factors/GBE) | Supervised (full/MCC) | Supervised (full/GBE) | 2-step PUL | SSPUL (MCC) | SSPUL (GBE) |  |
|  |  |  |  |  | (GBE) |  |  |  |
| BA | 0 | 0 | 0.02 | 0 | -0.02 | 0 | -0.02 |  |
|  | (-0.04, 0.04) | (-0.04, 0.04) | (-0.02, 0.07) | (-0.04, 0.04) | (-0.08, 0.02) | (-0.03, 0.03) | (-0.07, 0.03) |  |
| EO | -0.01 | 0 | 0.06 | 0 | -0.05 | 0.02 | -0.03 |  |
|  | (-0.09, 0.08) | (-0.10, 0.09) | (-0.03, 0.16) | (-0.08, 0.08) | (-0.17, 0.06) | (-0.05, 0.08) | (-0.13, 0.07) |  |
| Precision | -0.01 | -0.01 | -0.1 | 0.01 | -0.03 | -0.06 | -0.04 |  |
|  | (-0.07, 0.06) | (-0.09, 0.07) | (-0.19, 0.00) | (-0.09, 0.11) | (-0.12, 0.07) | (-0.15, 0.02) | (-0.13, 0.06) |  |
| Specificity | 0.01 | 0 | -0.01 | 0 | 0 | -0.02 | -0.01 |  |
|  | (-0.02, 0.04) | (-0.03, 0.04) | (-0.03, 0.00) | (-0.02, 0.03) | (-0.02, 0.02) | (-0.04, 0.00) | (-0.03, 0.01) |  |
| GBE | -0.01 | 0.01 | 0.15 | -0.01 | -0.01 | 0.13 | 0.01 |  |
|  | (-0.28, 0.30) | (-0.28, 0.31) | (0.02, 0.31) | (-0.22, 0.24) | (-0.26, 0.18) | (-0.04, 0.32) | (-0.15, 0.25) |  |
|  | | | | | | | |  |
| Metrics reported are means of differences between HL and NH-white for 1000 random test sets with 95% CI. Cutoffs for MCC-optimized models were selected by maximizing the MCC for unlabeled data in the validation set using proxy labels. Cutoff for GBE-optimized models were selected by optimizing the GBE for each race/ethnicity in the validation set. BA=balanced accuracy, EO=equal opportunity, GBE=group benefit equality, HL=Hispanic Latino, MCC=Matthew's correlation coefficient, NH-white=non-Hispanic white, NPV = negative predictive value, PUL=positive unlabeled learning, SSPUL=semi-supervised positive unlabeled learning. | | | | | | | |  |

| **Supplementary Table 6: Comparison of test set fairness with respect to EA and NH-white using MCC or GBE cutoffs** | | | | | | |  |  |
| --- | --- | --- | --- | --- | --- | --- | --- | --- |
|  |  |  |  |  |  |  |  |  |
| Metric | Supervised (risk factors/MCC) | Supervised (risk factors/GBE) | Supervised (full/MCC) | Supervised (full/GBE) | 2-step PUL | SSPUL (MCC) | SSPUL (GBE) |  |
|  |  |  |  |  | (GBE) |  |  |  |
| BA | -0.01 | 0 | -0.03 | -0.02 | -0.01 | -0.04 | -0.02 |  |
|  | (-0.05, 0.04) | (-0.04, 0.04) | (-0.07, 0.01) | (-0.07, 0.02) | (-0.06, 0.04) | (-0.08, 0.00) | (-0.07, 0.04) |  |
| EO | -0.03 | -0.01 | -0.07 | -0.04 | -0.02 | -0.09 | -0.03 |  |
|  | (-0.11, 0.06) | (-0.10, 0.09) | (-0.15, 0.03) | (-0.13, 0.05) | (-0.12, 0.08) | (-0.17, -0.01) | (-0.13, 0.08) |  |
| Precision | 0.02 | 0.01 | 0.02 | -0.03 | -0.02 | 0.02 | -0.03 |  |
|  | (-0.04, 0.10) | (-0.07, 0.09) | (-0.06, 0.10) | (-0.13, 0.07) | (-0.12, 0.06) | (-0.05, 0.09) | (-0.12, 0.07) |  |
| Specificity | 0.02 | -0.01 | 0 | -0.01 | -0.01 | 0.01 | -0.01 |  |
|  | (-0.02, 0.05) | (-0.05, 0.03) | (-0.01, 0.01) | (-0.04, 0.02) | (-0.03, 0.01) | (-0.01, 0.02) | (-0.03, 0.01) |  |
| GBE | -0.20 | 0 | -0.09 | 0 | 0.01 | -0.13 | 0 |  |
|  | (-0.46, 0.06) | (-0.29, 0.29) | (-0.20, 0.03) | (-0.24, 0.24) | (-0.17, 0.19) | (-0.25, -0.01) | (-0.17, 0.14) |  |
|  | | | | | | | |  |
| Metrics reported are means of differences between HL and NH-white for 1000 random test sets with 95% CI. Cutoffs for MCC-optimized models were selected by maximizing the MCC for unlabeled data in the validation set using proxy labels. Cutoff for GBE-optimized models were selected by optimizing the GBE for each race/ethnicity in the validation set. BA=balanced accuracy, EA=East Asian, EO=equal opportunity, GBE=group benefit equality, MCC=Matthew's correlation coefficient, NH-white=non-Hispanic white, NPV = negative predictive value, PUL=positive unlabeled learning, SSPUL=semi-supervised positive unlabeled learning. | | | | | | | |  |

**Supplementary Table 7: Sensitivity Analysis of Proxy Label Distribution Shifts**

|  | NH-white | | NH-AfAm | | HL | | EA | |
| --- | --- | --- | --- | --- | --- | --- | --- | --- |
| Proxy subset excluding: | Sensitivity | Precision | Sensitivity | Precision | Sensitivity | Precision | Sensitivity | Precision |
|  |  |  |  |  |  |  |  |  |
| All Meds | 0.82 | 0.82 | 0.83 | 0.79 | 0.79 | 0.78 | 0.79 | 0.81 |
|  | (0.70, 0.92) | (0.70, 0.92) | (0.64, 0.96) | (0.62, 0.93) | (0.60, 0.94) | (0.60, 0.93) | (0.63, 0.89) | (0.68, 0.92) |
| One ICD at a time |  |  |  |  |  |  |  |  |
| R41.1 | 0.8 | 0.8 | 0.81 | 0.78 | 0.77 | 0.77 | 0.75 | 0.79 |
|  | (0.71, 0.87) | (0.71, 0.87) | (0.65, 0.93) | (0.62, 0.92) | (0.60, 0.89) | (0.61, 0.90) | (0.59, 0.83) | (0.69, 0.92) |
| R41.2 | 0.8 | 0.8 | 0.81 | 0.78 | 0.77 | 0.76 | 0.75 | 0.8 |
|  | (0.71, 0.87) | (0.71, 0.87) | (0.65, 0.93) | (0.63, 0.91) | (0.60, 0.89) | (0.61, 0.89) | (0.60, 0.83) | (0.69, 0.92) |
| G31.01 | 0.8 | 0.8 | 0.81 | 0.78 | 0.77 | 0.77 | 0.75 | 0.8 |
|  | (0.71, 0.87) | (0.71, 0.87) | (0.65, 0.93) | (0.63, 0.92) | (0.60, 0.89) | (0.61, 0.90) | (0.60, 0.83) | (0.69, 0.92) |
| F01.518 | 0.8 | 0.8 | 0.81 | 0.78 | 0.77 | 0.77 | 0.75 | 0.79 |
|  | (0.71, 0.87) | (0.71, 0.87) | (0.65, 0.93) | (0.63, 0.92) | (0.60, 0.89) | (0.61, 0.90) | (0.60, 0.83) | (0.69, 0.92) |
| G31.1 | 0.8 | 0.8 | 0.81 | 0.78 | 0.77 | 0.77 | 0.75 | 0.8 |
|  | (0.71, 0.87) | (0.71, 0.87) | (0.65, 0.93) | (0.63, 0.92) | (0.60, 0.89) | (0.61, 0.90) | (0.60, 0.83) | (0.69, 0.92) |
| F01.50 | 0.8 | 0.8 | 0.81 | 0.78 | 0.77 | 0.76 | 0.75 | 0.79 |
|  | (0.71, 0.87) | (0.70, 0.87) | (0.64, 0.93) | (0.63, 0.92) | (0.60, 0.90) | (0.61, 0.90) | (0.60, 0.83) | (0.69, 0.92) |
| G31.85 | 0.8 | 0.8 | 0.81 | 0.78 | 0.78 | 0.77 | 0.75 | 0.8 |
|  | (0.71, 0.87) | (0.71, 0.87) | (0.65, 0.93) | (0.63, 0.92) | (0.60, 0.90) | (0.61, 0.90) | (0.60, 0.83) | (0.69, 0.92) |
| F01.51 | 0.8 | 0.8 | 0.81 | 0.78 | 0.77 | 0.77 | 0.75 | 0.8 |
|  | (0.71, 0.87) | (0.71, 0.87) | (0.65, 0.93) | (0.63, 0.92) | (0.60, 0.89) | (0.61, 0.90) | (0.60, 0.83) | (0.69, 0.92) |
| F02.811 | 0.8 | 0.8 | 0.81 | 0.78 | 0.77 | 0.77 | 0.75 | 0.8 |
|  | (0.71, 0.87) | (0.71, 0.87) | (0.65, 0.93) | (0.63, 0.92) | (0.60, 0.89) | (0.61, 0.90) | (0.60, 0.83) | (0.69, 0.92) |
| F03.918 | 0.8 | 0.8 | 0.81 | 0.78 | 0.77 | 0.77 | 0.75 | 0.79 |
|  | (0.71, 0.87) | (0.71, 0.87) | (0.65, 0.93) | (0.63, 0.92) | (0.60, 0.89) | (0.61, 0.90) | (0.60, 0.83) | (0.69, 0.92) |
| G31.84 | 0.8 | 0.8 | 0.81 | 0.78 | 0.77 | 0.76 | 0.76 | 0.79 |
|  | (0.68, 0.88) | (0.69, 0.89) | (0.64, 0.95) | (0.62, 0.92) | (0.59, 0.91) | (0.60, 0.90) | (0.61, 0.84) | (0.68, 0.91) |
| F03.90 | 0.76 | 0.76 | 0.76 | 0.73 | 0.73 | 0.72 | 0.72 | 0.75 |
|  | (0.64, 0.85) | (0.65, 0.85) | (0.58, 0.92) | (0.57, 0.88) | (0.55, 0.88) | (0.56, 0.86) | (0.56, 0.82) | (0.64, 0.86) |
| F03.911 | 0.8 | 0.8 | 0.81 | 0.78 | 0.77 | 0.77 | 0.75 | 0.8 |
|  | (0.71, 0.87) | (0.71, 0.87) | (0.65, 0.93) | (0.63, 0.92) | (0.60, 0.89) | (0.61, 0.90) | (0.60, 0.83) | (0.69, 0.92) |
| F03.91 | 0.8 | 0.8 | 0.81 | 0.78 | 0.77 | 0.77 | 0.75 | 0.79 |
|  | (0.70, 0.87) | (0.70, 0.87) | (0.64, 0.93) | (0.63, 0.91) | (0.60, 0.89) | (0.61, 0.90) | (0.60, 0.83) | (0.69, 0.92) |
| F02.818 | 0.8 | 0.8 | 0.81 | 0.78 | 0.77 | 0.77 | 0.75 | 0.8 |
|  | (0.71, 0.87) | (0.71, 0.87) | (0.65, 0.93) | (0.63, 0.92) | (0.60, 0.89) | (0.61, 0.90) | (0.60, 0.83) | (0.69, 0.92) |
| F01.511 | 0.8 | 0.8 | 0.81 | 0.78 | 0.77 | 0.77 | 0.75 | 0.8 |
|  | (0.71, 0.87) | (0.71, 0.87) | (0.65, 0.93) | (0.63, 0.92) | (0.60, 0.89) | (0.61, 0.90) | (0.60, 0.83) | (0.69, 0.92) |
| F02.80 | 0.8 | 0.8 | 0.81 | 0.78 | 0.77 | 0.77 | 0.75 | 0.8 |
|  | (0.71, 0.87) | (0.71, 0.87) | (0.65, 0.93) | (0.63, 0.92) | (0.60, 0.89) | (0.61, 0.90) | (0.60, 0.83) | (0.69, 0.92) |
| G31.83 | 0.8 | 0.8 | 0.81 | 0.78 | 0.77 | 0.77 | 0.75 | 0.8 |
|  | (0.71, 0.87) | (0.71, 0.87) | (0.65, 0.93) | (0.63, 0.92) | (0.60, 0.89) | (0.61, 0.90) | (0.60, 0.83) | (0.69, 0.92) |
| G31.09 | 0.8 | 0.8 | 0.81 | 0.78 | 0.77 | 0.77 | 0.75 | 0.8 |
|  | (0.71, 0.87) | (0.71, 0.87) | (0.65, 0.93) | (0.63, 0.92) | (0.60, 0.89) | (0.61, 0.90) | (0.60, 0.83) | (0.69, 0.92) |
| F02.81 | 0.8 | 0.8 | 0.81 | 0.78 | 0.77 | 0.77 | 0.75 | 0.8 |
|  | (0.71, 0.87) | (0.71, 0.87) | (0.65, 0.93) | (0.63, 0.92) | (0.60, 0.89) | (0.61, 0.90) | (0.60, 0.83) | (0.69, 0.92) |
| R41.3 | 0.64 | 0.64 | 0.69 | 0.68 | 0.61 | 0.61 | 0.59 | 0.59 |
|  | (0.58, 0.69) | (0.58, 0.69) | (0.54, 0.82) | (0.54, 0.83) | (0.48, 0.74) | (0.47, 0.73) | (0.47, 0.72) | (0.46, 0.73) |
| Phecodes (grouped ICDs) | | |  |  |  |  |  |  |
| 290.16 | 0.8 | 0.8 | 0.81 | 0.78 | 0.77 | 0.76 | 0.75 | 0.79 |
|  | (0.71, 0.87) | (0.70, 0.87) | (0.64, 0.93) | (0.63, 0.92) | (0.60, 0.90) | (0.61, 0.90) | (0.60, 0.83) | (0.69, 0.92) |
| 290.12 | 0.8 | 0.8 | 0.81 | 0.78 | 0.77 | 0.77 | 0.75 | 0.8 |
|  | (0.71, 0.87) | (0.71, 0.87) | (0.65, 0.93) | (0.63, 0.92) | (0.60, 0.89) | (0.61, 0.90) | (0.60, 0.83) | (0.69, 0.92) |
| 292.3 | 0.64 | 0.64 | 0.69 | 0.68 | 0.61 | 0.61 | 0.59 | 0.59 |
|  | (0.58, 0.69) | (0.58, 0.69) | (0.54, 0.82) | (0.53, 0.83) | (0.48, 0.74) | (0.47, 0.73) | (0.47, 0.71) | (0.46, 0.73) |
| 290.1 | 0.75 | 0.75 | 0.75 | 0.71 | 0.72 | 0.71 | 0.72 | 0.74 |
|  | (0.62, 0.85) | (0.63, 0.84) | (0.57, 0.92) | (0.54, 0.86) | (0.53, 0.88) | (0.54, 0.85) | (0.55, 0.81) | (0.62, 0.85) |
| 5 random ICDs at a time | |  |  |  |  |  |  |  |
| random1 | 0.6 | 0.6 | 0.67 | 0.65 | 0.56 | 0.56 | 0.57 | 0.57 |
|  | (0.54, 0.65) | (0.54, 0.65) | (0.49, 0.83) | (0.47, 0.81) | (0.43, 0.69) | (0.42, 0.70) | (0.45, 0.70) | (0.42, 0.70) |
| random2 | 0.8 | 0.8 | 0.81 | 0.78 | 0.77 | 0.77 | 0.75 | 0.8 |
|  | (0.71, 0.87) | (0.70, 0.87) | (0.65, 0.93) | (0.63, 0.92) | (0.60, 0.89) | (0.61, 0.90) | (0.60, 0.83) | (0.69, 0.92) |
| random3 | 0.8 | 0.8 | 0.81 | 0.78 | 0.77 | 0.76 | 0.75 | 0.79 |
|  | (0.70, 0.87) | (0.70, 0.87) | (0.64, 0.93) | (0.63, 0.91) | (0.60, 0.90) | (0.61, 0.90) | (0.60, 0.83) | (0.69, 0.92) |
| random4 | 0.8 | 0.8 | 0.81 | 0.78 | 0.77 | 0.77 | 0.75 | 0.79 |
|  | (0.71, 0.87) | (0.71, 0.87) | (0.65, 0.93) | (0.63, 0.92) | (0.60, 0.89) | (0.61, 0.90) | (0.60, 0.83) | (0.69, 0.92) |
| random5 | 0.76 | 0.76 | 0.76 | 0.73 | 0.73 | 0.72 | 0.72 | 0.75 |
|  | (0.64, 0.85) | (0.65, 0.85) | (0.58, 0.92) | (0.57, 0.88) | (0.55, 0.88) | (0.55, 0.86) | (0.57, 0.82) | (0.63, 0.86) |
|  |  |  |  |  |  |  |  |  |
| 290.16 consists of the ICDs F01.50, F01.51, F01.511, and F01.518. 290.12 consists of the ICDs G31.01, G31.09, and G31.83. 292.3 consists of the ICDs R41.1, R41.2, and R41.3. 290.1 consists of the ICDs F03.90, F03.91, F03.911, F03.918, F02.80, F02.81, F02.811, and F02.818. random1 consists of the ICDs G31.84, F01.518, G31.83, F03.918, and R41.3. random2 consists of the ICDs F03.911, F02.818, F02.80, F01.51, and G31.1. random3 consists of the ICDs F03.918, F02.80, R41.2, G31.09, and F03.91. random4 consists of the ICDs G31.83, F02.811, F03.911, F02.818, and F01.518. random5 consists of the ICDs G31.85, F01.51, F03.90, G31.83, and F03.918. EA=East Asian, HL=Hispanic Latino, ICD=International Classification of Diseases, NH=non-Hispanic, NH-AfAm=non-Hispanic African American. | | | | | | | | |

| **Supplementary Table 8:** **Classifier selection for baseline models and each step of SSPUL** | | | |
| --- | --- | --- | --- |
|  | Baseline Models & Step 1 of SSPUL | Step 2 of SSPUL | Step 3 of SSPUL |
|  | Mean AUCPR (5-Fold Cross-Validation, Train Set) | Mean AUCPR (Train Set) | Mean AUCPR (Validation Set) |
| Generalized Linear Model | 0.70 (0.69, 0.71) | 0.61 (0.60, 0.62) | 0.90 (0.87, 0.93) |
| Gradient Boosting Machine | 0.69 (0.68, 0.70) | 0.48 (0.43, 0.54) | 0.89 (0.84, 0.93) |
| XGBoost | 0.67 (0.67, 0.68) | 0.61 (0.59, 0.63) | 0.91 (0.87, 0.94) |
| Distributed Random Forest | 0.65 (0.64, 0.66) | 0.76 (0.74, 0.79) | 0.89 (0.86, 0.92) |
| The classifiers for the baseline models (Generalized Linear Model) and SSPUL steps (Generalized Linear Model, Distributed Random Forest, XGBoost for steps 1, 2, and 3, respectively) were selected based on the best average AUCPR, evaluated on training or validation sets from 10 initial splits. Oversampling of the minority class was implemented prior to training to address class imbalance. For both baseline models and steps 1 and 2 of SSPUL, the minority class was labeled positives. For step 3 of SSPUL, the minority class was labeled positives + additional (pseudo-labeled) positives. | | | |
